# The impact of injury severity and age on short-and long-term mortality and hospital length of stay after surgical stabilisation of rib fractures (SSRF): a German population-based propensity-score matched investigation

**DOI:** 10.1186/s13017-026-00682-2

**Published:** 2026-03-02

**Authors:** Michael David Huelskamp, Martial Mboulla Nzomo, Nicolas Horst, Laura Acar, Helena Duesing, Ursula Marschall, Michael Johannes Raschke, Steffen Rosslenbroich

**Affiliations:** 1 Department for Trauma-, Hand and Reconstructive Surgery, University Hospital Muenster, Muenster, Germany; 2 BARMER, Wuppertal, Germany; 3 Department for Trauma-, Hand and Reconstructive Surgery, Niels-Stensen-Kliniken, Marienhospital Osnabrueck, Osnabrueck, Germany

**Keywords:** Surgical stabilisation of rib fractures, SSRF, Chest trauma, Serial rib fractures, Rib fractures, Flail chest

## Abstract

**Background:**

Surgical stabilisation of rib fractures (SSRF) is a procedure that has been shown to reduce mortality and complications in patients with thoracic trauma. However, correct patient selection is required, since the immunological hit of a thoracic surgery in the context of trauma is not without risk. In this study we aimed to analyse real-world data on the effect of SSRF mortality and hospital length of stay.

**Methods:**

A retrospective propensity matched analysis of real-world health claims data of a German statutory health insurance company of Management of serial rib fractures and unstable thorax via SSRF compared to conservative treatment was performed. The primary endpoints were in-hospital mortality, 1 year post hospital mortality and intensive-care unit length of stay. Subgroup analyses with respect to age-group, overall injury severity and time-point of surgery were performed. Multivariate regression was used to confirm these results and analyse interaction between different variables.

**Results:**

Overall, 62,011 patients with serial rib fractures were identified, of which 532 patients receiving SSRF and 532 receiving conservative management could be matched. The data showed a significant reduction in in-hospital mortality in the SSRF cohort (4.7% for SSRF versus 9.21% for conservative treatment, *p* = 0.005). Subgroup analysis showed this effect to be most pronounced in patients with high, but not extremely high injury severity (injury severity score (ISS) 16–24). The survival benefit could be demonstrated across age groups, although the extent of the benefit decreased with increasing age. Hospital length of stay was increased in the SSRF cohort, although this effect was driven through an increase in the low injury severity (ISS < 16 subgroup).

**Conclusions:**

Overall, the data shows that SSRF can lead to reduced mortality in appropriately selected patients. Based on this real-world data, patients across all age-groups with high but not extreme injury severity (ISS 16–24), in other words patients in whom the thoracic injury is a major component of overall injury severity, appear to benefit most.

**Supplementary Information:**

The online version contains supplementary material available at 10.1186/s13017-026-00682-2.

## Introduction

Rib fractures are among the most frequent injuries in trauma patients and represent a significant source of morbidity and mortality, especially in elderly individuals and patients with multiple comorbidities. In a recent Dutch population-based study, rib fractures were present in 6% of hospitalised trauma patients, with an overall 30 days mortality of 6,9%, rising to 11,7% in patients aged ≥ 65 years [1]. These injuries impair respiratory function, predispose to pneumonia, and prolong hospitalisation [2, 3].

Traditionally, treatment was limited to analgesia and ventilatory support. Over the past two decades, surgical stabilisation of rib fractures (SSRF) has emerged as an evidence-based intervention, especially in flail chest and patients with refractory pain [4, 5]. Randomised trials and meta-analyses show that SSRF can reduce ventilator days, intensive care unit (ICU) length of stay, and pneumonia rates [3, 6–9]. Accordingly, recent CWIS/WSES consensus statements recommend SSRF in selected cases of chest wall instability and respiratory failure [10].

However, uncertainties remain regarding optimal timing and patient selection. While several studies suggest benefits of early surgery (< 72 h) [11–14], evidence is inconsistent, and long-term outcomes are insufficiently studied [15, 16].

Moreover, variability in practice is striking. A recent multicentre study by Hylands et al. analysed > 23,000 patients with flail chest and found wide differences in SSRF use across centres, without consistent survival benefit and with longer ICU and hospital stays in high-utilisation centres [17]. These findings emphasise that benefits of SSRF may depend strongly on patient characteristics rather than broad application.

Building on this, our study investigates the effect of SSRF on in-hospital and medium-term outcomes in a large national cohort. By examining the interaction between treatment, injury severity, and age, we aim to identify those patients most likely to benefit and to provide evidence that may help refine current indications for SSRF.

By analysing one of the largest retrospective, propensity score–matched cohorts based on nationwide statutory health insurance claims, our study captures real-world treatment patterns across all hospital levels. Thereby our study may reflect routine clinical practice more comprehensively than previous registry-based or centre-specific retrospective studies, which are often limited by centre selection or incomplete information on comorbidities in registries [18–21].

## Materials and methods

### Study design and inclusion criteria

This study is a retrospective, propensity-matched analysis based on data from the BARMER statutory health insurance company. Within the BARMER dataset all patients of ≥ 15 years of age with a diagnosis of a serial rib fracture, defined as a fracture of 3 or more ribs, or a mechanically unstable thorax identified by the International Classification of Disease and Related Health Problems, 10th Revision, German Modification (ICD-10-GM) codes listed in supplemental Table S1 between the years 2007 and 2022 were identified. S22.42 (fracture of two ribs) was excluded, since it does not conform to the definition of a serial rib fracture.

This rib fracture cohort was divided into intervention and control groups by the presence/absence of one of the procedure codes (*Operations- und Prozedurenschlüssel* (OPS)) for surgical stabilisation of rib fractures (SSRF) listed in supplemental Table S1 in the same hospital stay. Follow-up was defined from the date of index admission until death, end of insurance coverage, or up to 1 year post discharge, whichever occurred first. For both cohorts age, sex, all ICD and OPS codes from the index hospital stay and all ICD codes from the past year, duration between hospital admission and a possible SSRF surgery, mortality, hospital length of stay, duration of mechanical ventilation, incidence of pneumonia (defined by the ICD-codes in supplemental Table S1) within 1 year from the index admission and the case costs were extracted.

In-hospital all-cause mortality, 1 year all-cause mortality and hospital length of stay (HLOS) were defined as primary outcomes. Furthermore, duration of mechanical ventilation, case costs and the incidence of pneumonia up to 1 year after the injury were defined as secondary outcomes. The inclusion and exclusion criteria are summarised in the patients, interventions, controls and outcomes (PICO) statement below.

Since overall injury severity, patient age, and time to surgery are known to influence outcomes after SSRF, we performed subgroup analyses of these factors with respect to the primary endpoints [18, 22, 23]. For ISS subgroup analysis the cohort three strata were considered. These were defined as moderate (ISS ≤ 15), severe (ISS 16–24) and very severe (ISS ≥ 25) injury, as has previously been used [24]. For subgroup analysis with respect to age three strata were considered. These were defined as adults (18–64 years), elderly (65–79 years), oldest old (≥ 80 years). Finally, for subgroup analysis with respect to the timepoint of surgery within only the SSRF cohort early surgery was defined as within ≤ 72 h of injury and late surgery was defined as surgery after > 72 h after injury in accordance with previous studies and recommendations [6, 25].

### PICO statement

- ***Patients*** Patients with ICD-10-GM code of a serial rib fracture (S22.41, S22.43, S22.44) or mechanically unstable thorax (S22.5) within the time period 2007–2022 of age ≥ 15 years in the BARMER dataset (see supplemental Table S1 for details).
- ***Interventions*** Surgical stabilisation of rib fractures (SSRF) by any technique as identified by the OPS codes 5-346.5, 5-346.c0, 5-346.c1, 5-346.c2, 5-346.c3, 5-346.d0, 5-346.d1, 5-346.d2 and 5-346.d3 (see supplemental Table S1 for details).
- ***Controls*** Any treatment not including SSRF, i.e. absence of an SSRF OPS code.
- ***Outcomes*** Primary: in-hospital mortality, 1-year mortality, hospital length of stay (HLOS); secondary: duration of mechanical ventilation, case costs and 1-year pneumonia incidence.

A preliminary analysis of in-hospital mortality showed a high incidence of mortality in the conservative cohort within 48 h of admission, which may indicate that this cohort included patients that were too severely injured to be considered for SSRF or that were treated according to a palliative treatment concept, which would not be expected to be the case in the SSRF cohort. Therefore, all patients that died within 48 h of hospital admission within the conservative cohort only were excluded from further analysis, to avoid this potential bias. Patients who died within 48 h of admission were not excluded in the SSRF cohort, in order to not mask intervention related mortality.

### BARMER dataset

The BARMER is the second largest statutory health insurance company in Germany. It provides a nationwide longitudinal health claims database covering both outpatient and inpatient sectors, with up to 8.3 million (approximately 10% of the German population). The database has been frequently used for health care related research and results have been published in multiple publications [26, 27].

### Statistical analysis

Data handling in the BARMER database was performed using SQL SERVER (Version 15.0.4335.1, Microsoft) and R Core Team (Version 4.3.1). To achieve baseline comparability between the compared groups 1:1 nearest-neighbour propensity score matching (PSM) without replacement was performed. Matching was exact on sex, age classes and Injury Severity Score (ISS) on a logistic regression model also including the Elixhauser comorbidity score, specific relevant comorbidities and relevant concomitant injuries, including lung contusion and laceration (S27.3), as covariates (see Love plot in supplementary Fig. 1 for variables). Distributional balance for distance and a Love plot of covariate balance were used to assess matching effectiveness.

Calculation of the ISS from in-patient coded ICD-10-GM codes was performed as previously reported [28]. Briefly, the ICD-10-GM codes were transcoded into ICD-10-CM (clinical modification) codes and then used to calculate the ISS using the *icdpicr* package in R [28]. Furthermore, all in- and outpatient ICD-10-GM codes from the period up to 1 year before the index hospital admission were used to calculate the Elixhauser Index as a general measure of comorbidity load using the classification table in supplemental Table S2) as has been previously described [29–31].

Multivariate Cox proportional hazard regression was also performed in R. To aid interpretability of hazard ratios (HR) age and ISS were centred on their population mean (67.1 and 16.3 respectively) for purpose of regression analysis, as has been recommended [32].Sensitivity analyses excluding patients with flail-chest injury morphologies (S22.5) as well as a sensitivity analysis excluding patients with pulmonary contusion and laceration (S27.3) were performed for the regression analysis, since these have been shown to strongly influence the outcome in thoracic trauma [33, 34]. Further statistical analysis and visualisation was performed using GraphPad Prism (Version 10.4.1, GraphPad Software LLC). Survival was analysed using Kaplan-Meier curves and the Mantel-Cox Log rank test. Group comparisons were visualised using truncated violin plots and evaluated with two-tailed Mann-Whitney U-tests or two-tailed Kruskal-Wallis tests, as dictated by the number of groups. Statistical analysis of contingency table data was performed using Fisher’s exact test. Statistical significance was defined as *p* ≤ 0.05. Effect sizes are given as odds ratio (OR) for incidence rates or hazard ratio (HR) for time to event analysis and as the difference between medians (MD) using the Hodges-Lehmann-estimator for continuous outcomes as dictated by the data type each with 95% confidence intervals (95% CI).

### Reporting and registration

This study was conducted in accordance with ethics approval as granted by the Ethikkommission Westfalen-Lippe (2024-006-f-S). All data is of a retrospective nature and was provided by the BARMER in anonymised fashion for further analysis. Reporting was performed according to the STROBE guidelines (see checklist in supplemental information).

## Results

The search of the BARMER database yielded 62,011 patients with an ICD-GM code for a serial rib fracture or an unstable thorax. Of these, 534 also had an OPS code for SSRF. The demographics of this overall cohort pre-matching are shown in Table 1.

### Matching and demographics

**Fig. 1:**
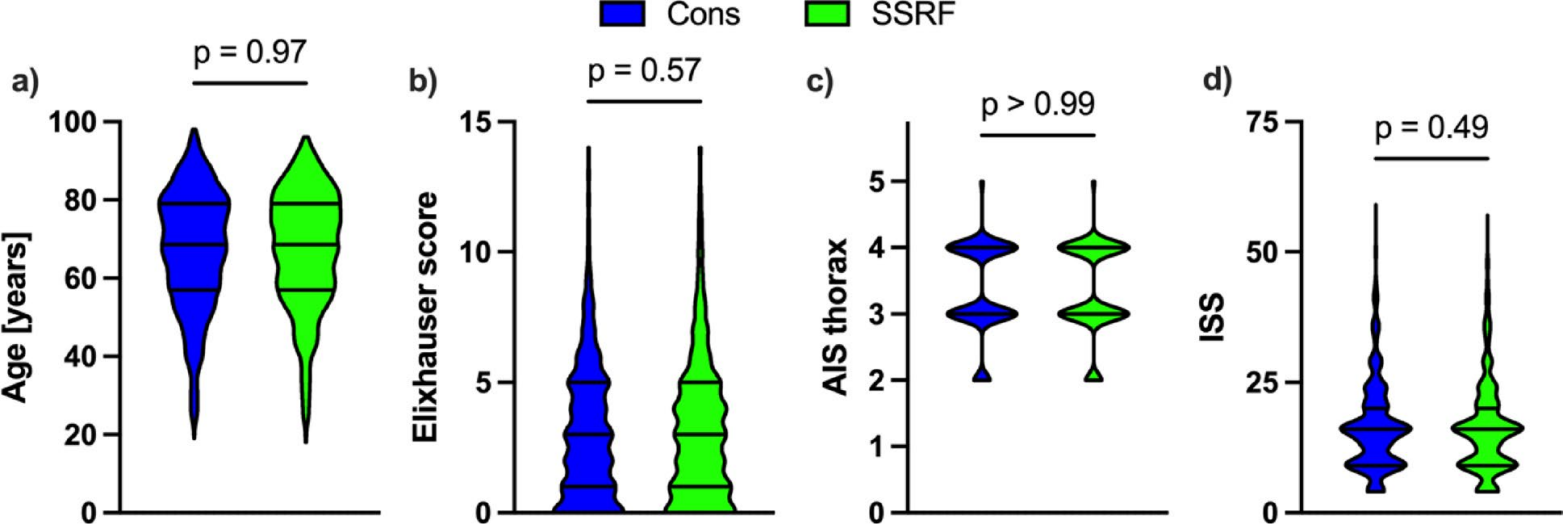
Distribution of demographic factors (age in years (**a**) and Elixhauser score (**b**)) and trauma severity (Abbreviated Injury Scale (AIS, **c**) and Injury Severity Score (ISS, **d**)) after matching. Shown are truncated violin plots with median and 1st and 3rd quartiles for conservative treatment (Cons, blue) and surgical stabilisation of rib fractures (SSRF, green). Statistical analysis was performed using two-tailed Mann-Whitney U-tests and *p* < 0.05 was considered significant. *AIS* abbreviated injury scale, *ISS* injury severity score

Matching was then performed to yield the post-matching cohort (Table 1). 532 patients treated conservatively (mean age 67.12 years, 15.1% female) and 532 patients treated with SSRF (mean age 67.12 years, 15.0% female) could be included for further analysis. There was no significant difference between the groups with respect to age, sex, Elixhauser score, thorax AIS or ISS, with distributions shown in Fig. 1. Distributional balance for distance and a Love plot for covariate balance are shown in supplemental Fig. S1.

**Table 1:** Demographic characteristics of pre- and post-matching cohorts

	Pre-matching		Post-matching 1:1		
	Conservative (*n* = 61,477)	SSRF (*n* = 534)	Conservative (*n* = 532)	SSRF (*n* = 532)	*p*-value
Female sex [n (%)]	30,739 (50.0)	198 (37.1)	197 (37.0)	197 (37.0)	> 0.99
Age [years, mean (SD)]	72.47 (16.1)	67.16 (15.0)	67.12 (15.1)	67.12 (15.0)	0.97
Elixhauser score [median (Q_1_ - Q_3_)]	5 (0–12)	2 (0–10)	3 (1–5)	3 (1–5)	0.57
Thorax AIS [median (Q_1_ - Q_3_)]	2 (2–3)	3 (3–4)	3 (3–4)	3 (3–4)	> 0.99
ISS [median (Q_1_ - Q_3_)]	8 (4–12)	16 (9–20)	16 (9–20)	16 (9–20)	0.49
Flail chest [n (%)]	1,242 (2.02)	534 (44.4)	232 (43.6)	235 (44.2)	0.90
Pulmonary contusion/laceration [n (%)]	5,032 (8.2)	176 (33.0)	169 (31.8)	176 (33.1)	0.69

Characteristics of the patient cohorts pre- and post-matching given as mean with standard deviation (SD) or as the number (n) with the percentage (%). Significance was calculated with two-tailed Mann-Whitney U-test and Fisher’s exact test as appropriate. *p* < 0.05 was considered statistically significant. *SSRF* surgical stabilisation of rib fractures, *AIS* abbreviated injury scale, *ISS* injury severity score, *SD* standard deviation.

### Primary outcomes

**Fig. 2:**
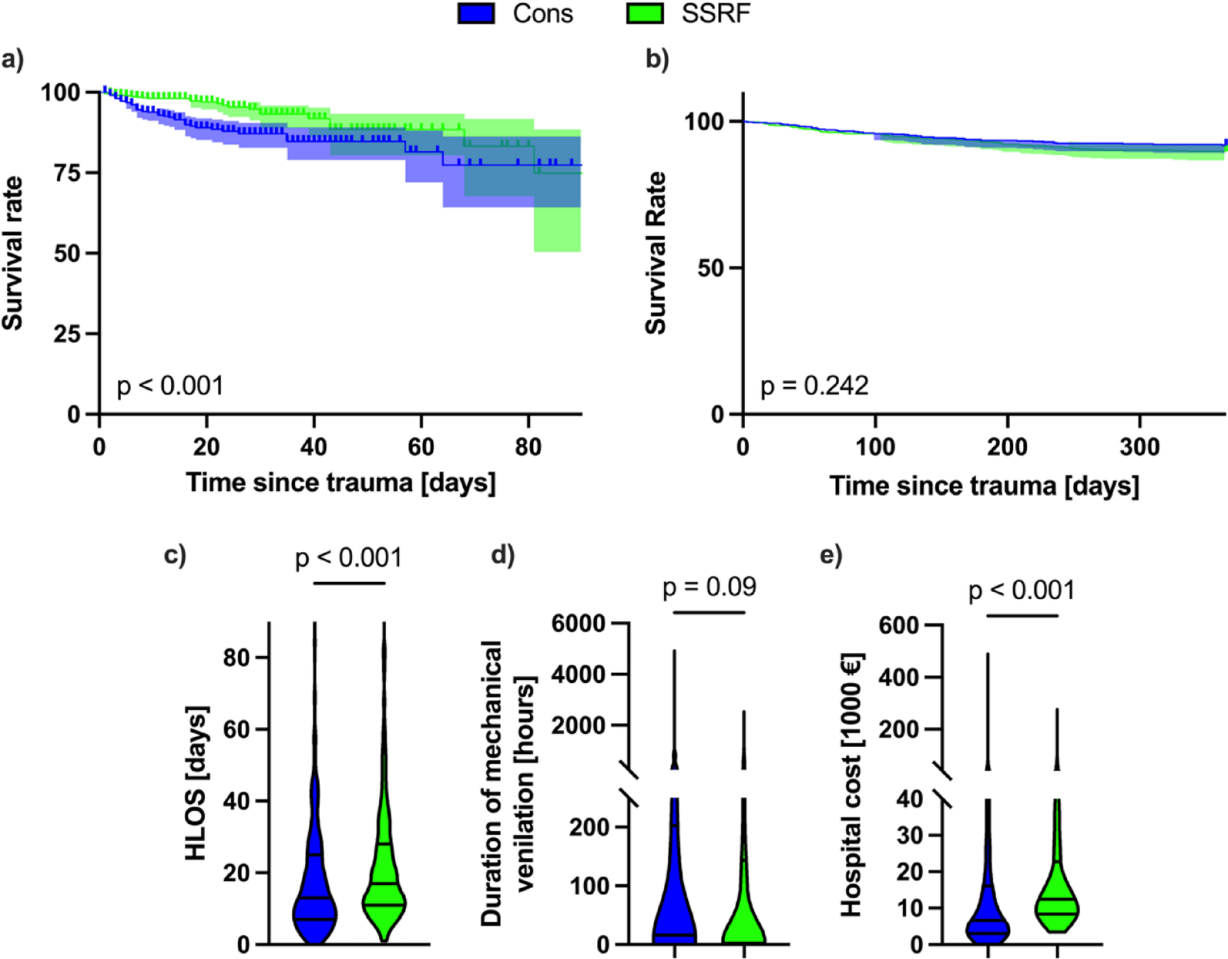
Outcomes after conservative treatment (Cons, blue) and surgical stabilisation of rib fractures (SSRF, green). Shown are Kaplan-Meier plots of survival rate to hospital discharge, excluding mortality within 48 h of admission in the conservative group truncated to 90 days (**a)** and 1-year mortality excluding in-hospital mortality (**b)** as well as truncated violin plots with median and 1st and 3rd quartiles of hospital length of stay (HLOS) in days (**c**), duration of mechanical ventilation in hours (**d**) and hospital costs in Euros (**e**). Statistical analysis was performed using Mantel-Cox Log rank test for survival analysis and Mann-Whitney U-tests as appropriate. *p* < 0.05 was considered to be statistically significant.

The rate of hospital mortality was 9.21% in the conservative cohort (*n* = 49 of 532) and 4.7% (*n* = 25 of 532) in the SSRF cohort. This difference was statistically significant with OR 2.057 (95% CI 1.263–3.372, *p* = 0.005) and is reflected in the Kaplan-Meier plot of survival rate to hospital discharge in Fig. 2a, which also showed a significant difference (HR 2.216, 95% CI 1.401–3.506, *p* < 0.001). The rate of 1-year mortality excluding hospital mortality was 8.07% (*n* = 39 of 483) in the conservative cohort and 10.26% (*n* = 52 of 507) in the SSRF cohort, which was not statistically significant (OR 0.769, 95% CI 0.497–1.191, *p* = 0.27). The median HLOS was 13 days in the conservative cohort and 17 days in the SSRF cohort (Fig. 1c), which was found to be statistically significant (MD 3, 95% CI 2–5, *p* < 0.001).

### Secondary outcomes

The median duration of mechanical ventilation was 16 h in the conservative cohort and 2 h in the SSRF cohort (Fig. 2d), which was not statistically significant (*p* = 0.09). However, there was a strong tendency towards reduced duration of mechanical ventilation in the SSRF cohort. The case costs for the initial hospital stay until first discharge were significantly lower in the conservative cohort (median 6,633€) than in the SSRF cohort (median 12,470 €) as is shown in Fig. 1e (MD 5.835, 95% CI 4.469–5.897, *p* < 0.001). The in-hospital pneumonia rate was 17.48% for conservative treatment and 17.67% for SSRF (OR 0.987, 95% CI 0.722–1.349, *p* > 0.99). The rate of pneumonia in the year following the trauma excluding the hospital stay was slightly but not significantly higher, in the conservative cohort compared to the SSRF cohort (7.45% versus 5.52%, OR 1.378, 95% CI 0.832–2.266, *p* = 0.25).

### Subgroup analysis of injury severity score (ISS)

**Fig. 3:**
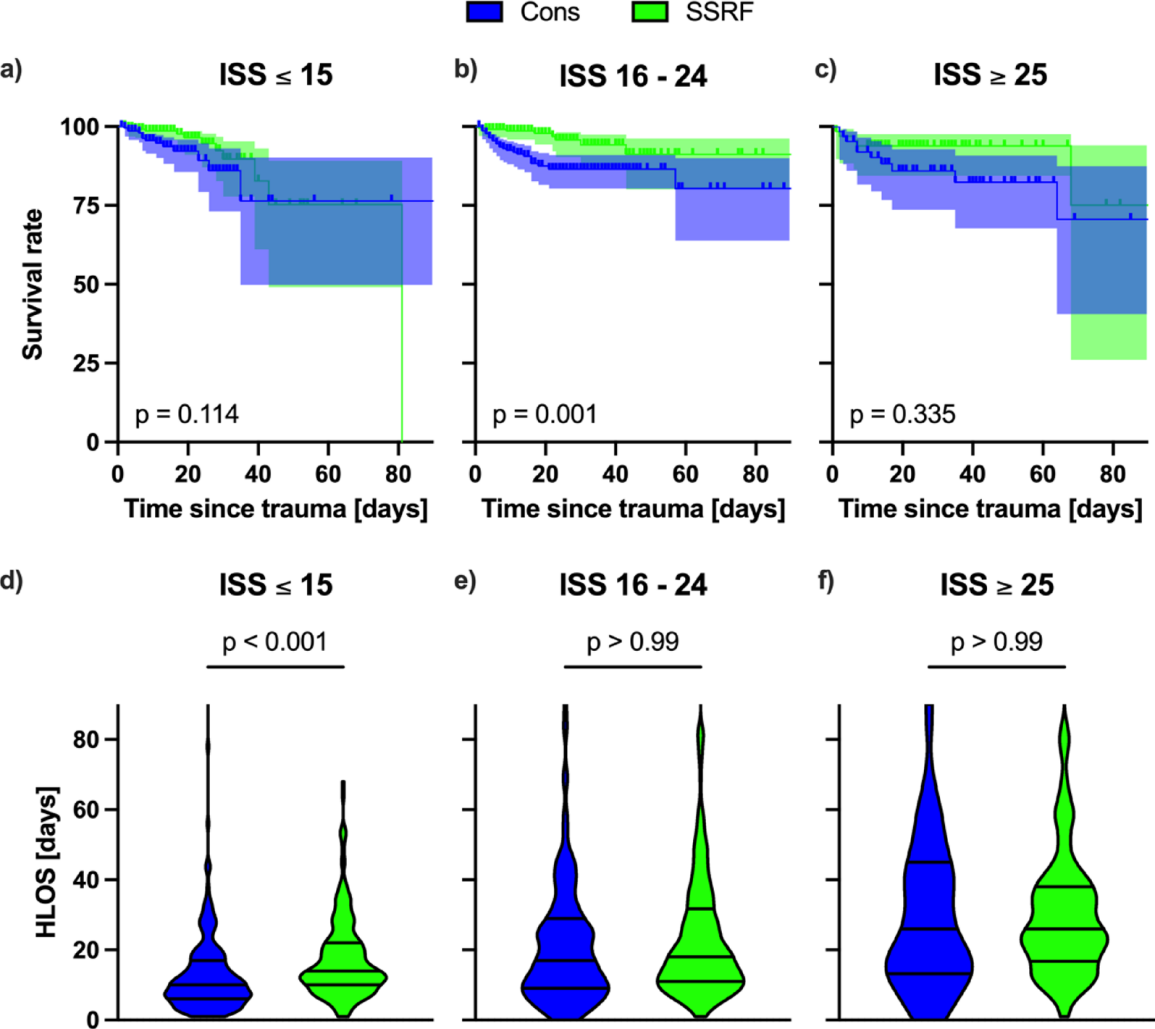
Subgroup analysis with respect to injury severity score (ISS) for conservative treatment (cons, blue) and surgical stabilisation of rib fractures (SSRF, green). Shown are Kaplan-Meier plots of survival rate to hospital discharge, excluding mortality within 48 h in the conservative cohort only, truncated at 90 days post admission (**a**–**c**) and truncated violin plots with median and interquartile ranges of hospital length of stay (HLOS) in days (**d** – **e**) for each of the subgroups ISS ≤ 15 (Cons n = 230; SSRF n = 230), ISS 16–24 (Cons n = 232; SSRF n = 236) and ISS ≥ 25 (Cons n = 64; SSRF n = 66). Statistical analysis was performed using Mantel-Cox Log rank test for survival analysis and Mann-Whitney U-tests as appropriate. *p* < 0.05 was considered to be statistically significant.

There were no significant differences between the demographics of the conservative and SSRF cohorts with respect to the ISS strata (moderate (ISS ≤ 15), severe (ISS 16–24) and very severe (ISS ≥ 25) injury). There was no significant difference between the Kaplan-Meier survival curves for moderate (Fig. 3a, HR 0.522, 95% CI 0.233–1.168, *p* = 0.11) or very severe injury (Fig. 3c, HR 0.616, 95% CI 0.229–1.651, *p* = 0.34), although there was a tendency towards improved survival in the SSRF group for both. However, a significant survival advantage was observed in patients treated with SSRF in the severe injury stratum (Fig. 3b, HR 0.321, 95% CI 0.162–0.636, *p* = 0.001). Within the moderate injury subgroup, the median HLOS was 10 days for conservative treatment and 14 days for SSRF (Fig. 3d, MD 4, 95% CI 3–6, *p* < 0.001). However, there was no significant difference between HLOS in severe (Fig. 3e, MD 2, 95% CI − 1–4, *p* = 0.35) or very severe (Fig. 3f, MD 0, 95% CI − 7–8, *p* > 0.99) strata.

### Subgroup analysis of age

**Fig. 4:**
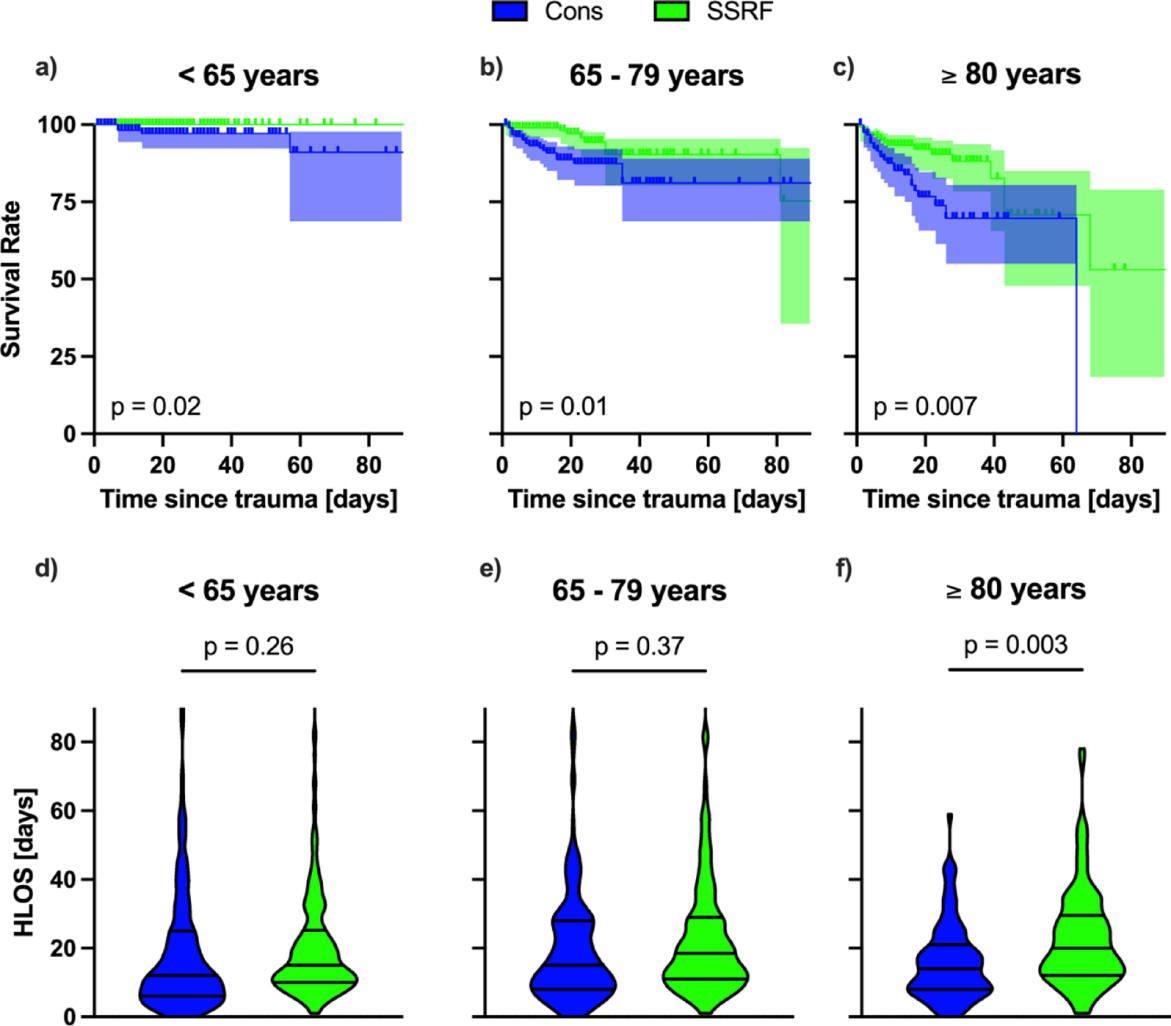
Subgroup analysis with respect age for conservative treatment (cons, blue) and surgical stabilisation of rib fractures (SSRF, green). Shown are Kaplan-Meier plots of survival rate to hospital discharge, excluding mortality within 48 h in the conservative cohort only, truncated at 90 days post admission (**a – c**) and truncated violin plots with median and interquartile ranges of hospital length of stay (HLOS) in days (**d – e**) for each of the subgroups < 65 years (Cons *n* = 209; SSRF *n* = 214), 65–79 years (Cons *n* = 173; SSRF *n* = 184) and ≥ 80 years (Cons *n* = 101; SSRF *n* = 214). Statistical analysis was performed using Mantel-Cox Log rank test for survival analysis and Kruskal-Wallis tests as appropriate. *p* < 0.05 was considered to be statistically significant

There were no significant differences between the demographics of the conservative and SSRF cohorts within each of the age strata. Kaplan-Meier survival curves differed across age strata, with mortality increasing with age. In the adult stratum all patients that received SSRF survived until hospital discharge, whilst 5 died in the conservative group (Fig. 4a, HR 0.123, 95% CI 0.021–0.730, *p* = 0.02). Furthermore, mortality was lower in the SSRF group in both the elderly (Fig. 4b, HR 0.398, 95% CI 0191–0.828, *p* = 0.01) and in the oldest-old strata (Fig. 4c, HR 1.453, 95% CI 0.209–0.776, *p* = 0.007). There was no significant difference in HLOS within the elderly stratum (Fig. 4e, MD 3, 95% CI 0–5, *p* = 0.07). However, HLOS was significantly longer in the SSRF group in the adult (Fig. 4d, MD 3, 95% CI 1–5, *p* = 0.05) and the oldest-old stratum (Fig. 4f, MD 6, 95% CI 3–9, *p* = 0.003).

### Regression analysis of survival to discharge

To further validate the results with respect to mortality rate in the overall cohort and in the ISS and age subgroups, a Cox proportional hazards regression of survival to discharge was performed using the type of treatment, sex, age centred on 67.1 years (age_c_), Elixhauser score, thorax AIS and ISS centred on 16.3 (ISS_c_) and interaction terms between the type of treatment and ISS_c_ as well as age_c_ as variables. The HRs with 95% CI thereof and p-values are shown in Table 2. The concordance of the regression model was 0.793, indicating its robust prediction characteristics.

The strongest predictor of mortality was the type of treatment with a HR of 0.211 (95% CI 0.087–0.514, *p* < 0.001). Furthermore, age_c_ was a significant predictor of mortality with a HR of 1.064 (95% CI 1.037–1.092, *p* < 0.001) indicating a 6.4% increased risk of mortality per year of age. Sex, thorax AIS, ISS_c_ and Elixhauser score did not show a significant effect, although there was a trend towards an increased risk of mortality at higher thorax AIS (HR = 1.404, 95% CI 0.890–2.215, *p* = 0.145).

The interaction terms between the type of treatment and age_c_ as well as ISS_c_ did not reach statistical significance. However, there was a tendency towards a lower survival advantage through SSRF at higher age indicated by a HR of 1.053 for the interaction term between these variables (SSRF: Age_c_, 95% CI 0.997–1.111, *p* = 0.063).

Sensitivity analysis was performed with respect to flail-chest and non-flail fracture morphology (S22.5) and the presence or absence of pulmonary contusion or laceration (S27.3). The results of these regression models are shown in supplementary tables S3 and S4 respectively. Generally, the direction of the observed effects remained constant. However, the benefit of SSRF was higher in the flail chest group (HR 0.137, 95 CI 0.035–0.5321, *p* = 0.004) than in the non-flail group (HR 0.343, 95% CI 0.089–1.312, *p* = 0. 118), the latter of which showed the same trend towards a positive effect of SSRF, but did not reach statistical significance. Conversely, the beneficial effect of SSRF was higher in the subgroup without pulmonary injury (HR 0.193, 95% CI 0.067–0.557, *p* = 0.002) than in the subgroup with pulmonary injury (HR 0.293, 95% CI 0.053–1.607, *p* = 0.157), the latter of which again showed a trend but did not reach statistical significance. The concordance of all sensitivity analysis regression models remained high at > 0.78, indicating robust prediction characteristics.

**Table 2:** Regression analysis of survival to hospital discharge

Variable	Hazard ratio	95% Confidence interval	*p*-value
SSRF	0.211	0.087–0.514	**< 0.001**
Female sex	0.724	0.451–1.163	0.182
Age_c_	1.064	1.037–1.092	**< 0.001**
Elixhauser score	1.058	0.981–1.141	0.143
Thorax AIS	1.404	0.890–2.215	0.145
ISS_c_	1.010	0.971–1.050	0.623
SSRF: Age_c_	1.053	0.997–1.111	0.063
SSRF: ISS_c_	1.009	0.948–1.073	0.778

Parameters of the Cox proportional hazard regression of survival to hospital discharge with respect to the treatment type (conservative versus surgical stabilisation of rib fractures (SSRF)), sex, age_c_ centred on 67.1 years, Elixhauser score, thorax abbreviated injury scale (AIS) and injury severity score (ISS_c_) centred on 16.1 as well as the interactions between SSRF and Age (SSRF: Age_c_) and between SSRF and ISS (SSRF: ISS_c_). Through centring of age and ISS the hazard ratio for SSRF is interpretable as the hazard ratio for a patient of age 67.1 years with ISS 16.3. Given are the hazard ratios, 95% confidence interval of the hazard ratio and *p*-values. *p* < 0.05 was considered statistically significant. Significant *p*-values are highlighted in bold font.

### Subgroup analysis of timepoint of surgery

**Fig. 5:**
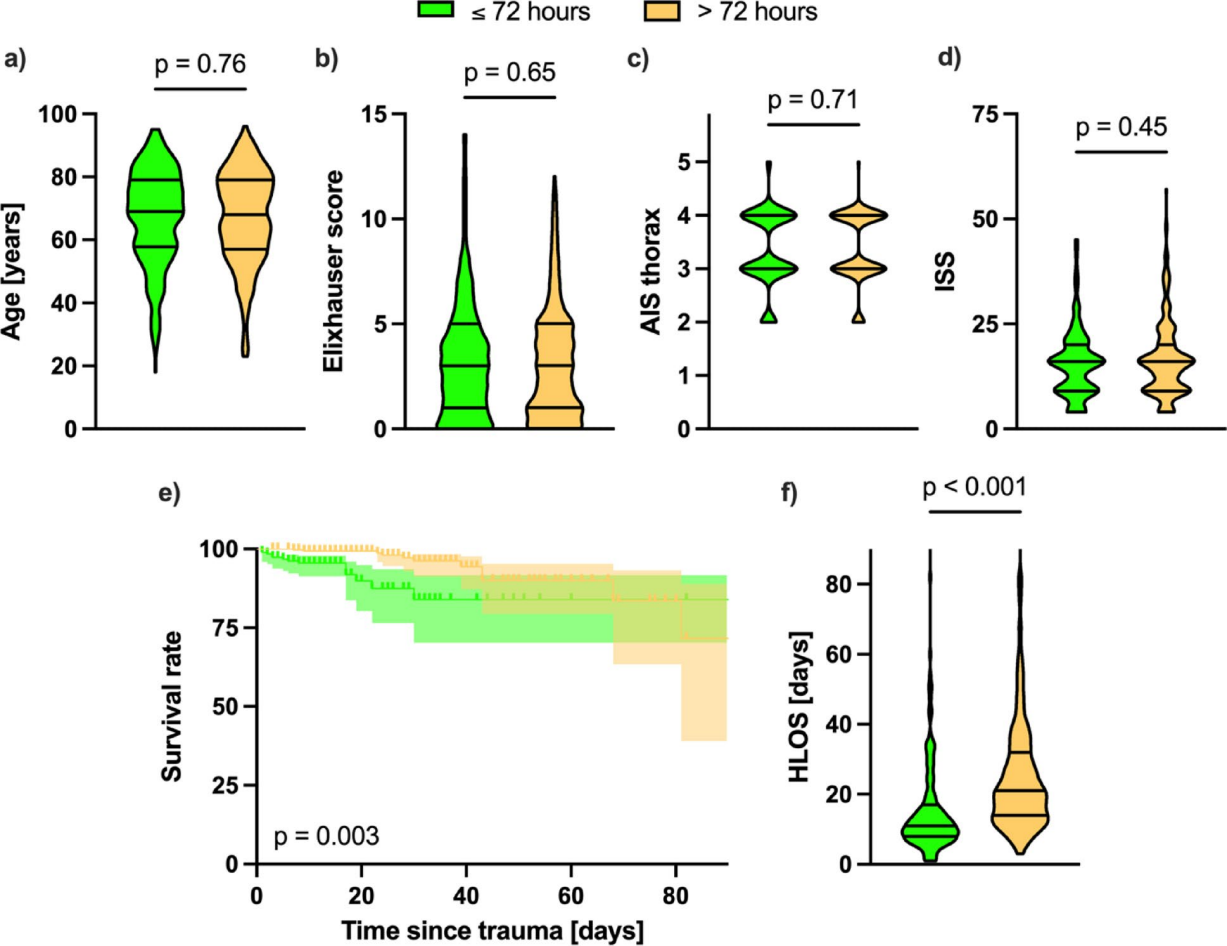
Subgroup analysis of the primary endpoints with respect to the timepoint of surgery within the surgical stabilisation of rib fractures (SSRF) cohort. Early surgery was defined as ≤ 72 h from trauma (green, *n* = 180) and late surgery was defined as > 72 h from trauma (orange, *n* = 327). **a–d** show the demographics of the two subgroups with respect to age, Elixhauser score, abbreviated injury scale (AIS) thorax and injury severity scale (ISS). **e** shows a Kaplan-Meier plot of survival to hospital discharged, truncated at 90 days post trauma. **f** shows the hospital length of stay (HLOS) as truncated violin plots with median as well as interquartile ranges. Statistical analysis was performed using Mantel-Cox Log rank test for survival analysis and Mann-Whitney U-tests as appropriate. *p* < 0.05 was considered to be statistically significant

Furthermore, subgroup analysis within the SSRF cohort was performed with respect to the timepoint of surgery, with early surgery defined as within 72 h of trauma and late surgery as occurring greater than 72 h after trauma. A total of 180 patients in this cohort received SSRF within 72 h and 327 received SSRF after more than 72 h. The demographic characteristics of these two subgroups (age, Elixhauser score, AIS thorax and ISS) are shown in Fig. 5a and d. There was no significant difference between the subgroup with respect to these parameters.

The overall mortality rate was 6.74% (13 of 193) in the early surgery group and 3.54% (12 of 339), which did not reach statistical significance (OR 1.968, 95% CI 0.895–4.450, *p* = 0.13). However, the Kaplan-Meier plot of survival rates in the two subgroups shows a significant difference between the two curves (Fig. 5e, HR 0.255, 95% CI 0.103–0.630, *p* = 0.003). In contrast, HLOS excluding cases of in-hospital mortality was significantly lower in the early surgery group with a median of 11 days (8–17) compared to a median of 21 days (14–32) in the late surgery subgroup (Fig. 5f, MD 8, 95% CI 7–10, *p* < 0.001).

In order to further validate the results of the subgroup analysis with respect to the timepoint of SSRF a further Cox proportional hazard regression of survival was performed using the time point of treatment, sex, age_c_, Elixhauser score, thorax AIS and ISS_c_ as variables. The HR with 95% CI thereof and p-values are shown in Table 3. The concordance of the regression model was 0.889 indicating its robust prediction characteristics. Late surgery was associated with a reduced risk of mortality (HR = 0.247, 95% CI 0.101–0.606, *p* = 0.002). Again, higher age was associated with an increased risk of mortality (HR 1.137, 95% CI 1.079–1.198, *p* < 0.001) and sex, thorax AIS, ISS and Elixhauser score did not show a significant effect.

**Table 3:** Regression analysis of survival to hospital discharge with respect to timepoint of surgery

Variable	Hazard ratio	95% Confidence interval	*p*-value
SSRF > 72 h	0.247	0.101–0.606	**0.002**
Female sex	0.617	0.259–1.469	0.275
Age_c_	1.137	1.079–1.198	**< 0.001**
Elixhauser score	0.991	0.872–1.127	0.890
Thorax AIS	1.246	0.570–2.723	0.581
ISS_c_	1.019	0.956–1.086	0.557

Parameters of the Cox proportional hazard regression of survival to hospital discharge with respect to the timepoint of surgical stabilisation of rib fractures (SSR, early defined as ≤ 72 hours after trauma and late defined as > 72 hours after trauma), sex, age_c_ centred on 67.1 years, Elixhauser score, thorax abbreviated injury scale (AIS) and injury severity score (ISS_c_) centred on 16.1. Through centring of age and ISS the hazard ratio for late SSRF is interpretable as the hazard ratio for a patient of age 67.1 years and ISS 16.3. Given are the hazard ratios, 95% confidence interval of the hazard ratio and *p*-values. *p* < 0.05 was considered statistically significant. Significant *p*-values are highlighted in bold font.

## Discussion

This population-based, propensity score-matched analysis demonstrates that SSRF is associated with a substantially lower in-hospital mortality compared to conservative management. In the matched cohort, mortality was reduced from 9.2% in the non-operative group to 4.7% in patients undergoing SSRF. These findings are consistent with previous randomised and observational studies reporting a survival benefit of SSRF (10,30,31). For instance, Dehghan et al. analysed more than 117,000 patients with chest wall trauma and showed that surgical stabilisation of flail chest was associated with a reduced risk of early mortality compared with conservative treatment [35]. Similarly, the CWIS-TBI multicentre study by Prins et al. demonstrated that SSRF in patients with rib fractures and traumatic brain injury reduced 30-day mortality, with benefits even in severe TBI (GCS ≤ 8) and without worsening neurological outcomes [36].

The survival benefit of SSRF was most pronounced in patients with flail chest. However, sensitivity analyses suggest that the observed beneficial effect may also extend to non-flail injury patterns, although statistical significance was not reached in this subgroup, possibly due to reduced sample size. This finding is particularly relevant, as the benefit of SSRF in flail chest has already been well established in the literature. Our results therefore indicate that, while flail chest patients have the greatest benefit, selected patients with non-flail serial rib fractures may also benefit from SSRF.

Not all studies, however, have demonstrated universal benefit. Hylands et al., in a multicentre study of more than 23,000 patients with flail chest, reported extreme variability in SSRF use across 354 trauma centres (0–88%). Importantly, hospitals with the highest SSRF rates did not show improved mortality but exhibited longer ICU and hospital stays, higher tracheostomy rates, and lower rates of discharge to home. Although instrumental variable analysis suggested a mortality benefit in selected patients, the authors emphasised the importance of precise indications and individualised decision-making rather than indiscriminate use of SSRF [17].

In a recent registry study, Lin et al. focused on functionally dependent patients with ≥ 3 rib fractures and thoracic AIS ≥ 3 and found a survival benefit of SSRF after propensity matching (4.8% vs. 8.7%), with early surgery reducing ventilation days, ICU stay, and hospital length of stay. Interestingly, late SSRF still conferred survival benefits compared to conservative management (3.8% vs. 10.9%) [37]. Our study not only complements but extends these findings. While Lin et al. focused primarily on functional dependency and timing, we provide nationwide real-world data across all hospital levels and uniquely demonstrate interaction effects of SSRF with injury severity and age, validated by regression analyses.

In our cohort, the strongest survival benefit was observed in patients with moderate trauma severity (ISS 16–24). Patients with minor injuries (ISS ≤ 15) and those with very severe trauma (ISS ≥ 25) did not derive a clear advantage, suggesting that SSRF is most effective when chest wall injury is a major contributor to outcome. This interpretation is supported by van Wijck et al., who found that patients with moderate pulmonary contusions undergoing SSRF required fewer ventilator days compared to non-operative management, whereas outcomes were similar in patients with severe contusions [33]. In the present study, pulmonary contusion or laceration were included as a covariate. In addition, sensitivity analyses with respect to pulmonary injury showed a consistent direction of effect for SSRF for both groups. However, the treatment effect was larger in the group without pulmonary injury and did not reach statistical significance in the group with pulmonary injury, which may be due to reduced sample size in the sensitivity analysis. These findings suggest that SSRF may provide a benefit both in the presence and absence of pulmonary injury, but that it may not be able to compensate for pulmonary impairment, which is not due to mechanical instability.

Importantly, the benefit of SSRF was not confined to younger patients. Even patients ≥ 80 years experienced reduced mortality compared with conservative management. These findings mirror earlier reports: Duong et al. showed that elderly patients undergoing SSRF had fewer pulmonary complications and shorter ICU stays despite higher overall mortality [38], while Zhang et al. demonstrated faster fracture healing and reduced analgesic requirements in elderly patients despite prolonged hospitalisation [19]. Our regression models confirmed age as an independent risk factor, with interaction terms indicating that the relative benefit of SSRF diminishes with increasing age. This result underscores that SSRF can be extended to older adults with careful selection, although the effect size decreases in advanced age.

These subgroup effects were further confirmed in multivariate regression analyses, underscoring the robustness of the observed associations. This analysis showed a beneficial effect of SSRF on survival after adjustment for age, however the interaction term between age and treatment indicated that the survival benefit of SSRF was larger in younger patients, which is in agreement with previously published studies [19, 31].

In contrast to mortality, SSRF was associated with longer hospital length of stay, with a median of 17 days compared to 13 days after conservative treatment. This prolongation was particularly evident in patients with minor trauma and in those ≥ 80 years. Prior studies have reported heterogeneous findings: Xiao et al. found no significant difference in HLOS, Wijffels et al. reported shorter stays after SSRF, and Long et al. observed reduced HLOS in their meta-analysis [7, 22, 39]. In our cohort, no significant HLOS difference was seen in patients with ISS ≥ 16, suggesting that in more severely injured patients’ hospital stay is determined largely by complications and associated injuries rather than the choice of rib fixation [18, 40].

The age-stratified analysis did reveal that SSRF patients aged ≥ 80 years had significantly longer HLOS than younger patients. While this may raise concern about postoperative recovery in this population, the result must be interpreted cautiously: age-related delays in mobilisation, comorbidity management, or discharge planning could have a significant influence on the duration. A prior study by Duong et al. showed that elderly patients undergoing SSRF did not experience increased complications, and prolonged hospitalisation may reflect non-surgical factors such as functional dependency or post-acute placement needs [38]. Zhang et al. found similar results, showing longer HLOS in elderly patients while still showing advantages in pain relief as well as time to fracture healing [19].

Timing analysis showed that early SSRF (≤ 72 h) was associated with shorter HLOS, consistent with previous work by Pieracci et al. and Zhu et al., who reported reductions in ventilator duration, pneumonia, ICU stay, and hospital stay with early fixation [11, 12, 14, 41]. Pieracci et al., while analysing prospectively collected SSRF data from databases 2006 to 2016, demonstrated that early SSRF shortened mechanical ventilation and reduced the risk of pneumonia as well as likelihood of tracheostomy [12]. In addition a significant longer operating time was recorded in comparison to early SSRF [12]. Chen Zhu et al. similarly found reduced HLOS, ICU LOS, shorter mechanical ventilation and pneumonia rates in geriatric trauma patients treated within 72 h [41].

While our data initially suggest that SSRF may prolong HLOS, especially in older or mildly injured patients, these effects appear heterogeneous and modifiable by surgical timing. As described above most studies support that, when performed early and in appropriate patients, SSRF does not increase and may even reduce HLOS [11, 12, 14, 41]. These findings underline the need for individualised patient selection and further studies to evaluate surgical timing on HLOS.

This may be in part due to the current indication practices for SSRF in Germany. Several studies have shown that the timing of SSRF in large German cohorts is to a large extent > 72 hours after trauma [20]. Furthermore, SSRF is still only performed rarely in Germany [20, 21]. It seems likely that early or immediate SSRF is mostly considered in patients with more severe injuries or physiological instability, which may however not be fully reflected in the health claims data. This confounding through selection of the more severely injured patients for early SSRF through clinical experience could explain the higher mortality rate in the early SSRF cohort. A more detailed analysis including extended physiological parameters over the course of days after the trauma would be necessary to further evaluate this possibility.

Furthermore, studies suggested that timing alone should not dictate the indication for SSRF. Instead, physiological parameters (e.g., oxygenation, pain control, pulmonary status) should guide the decision, especially in frail or elderly patients [34, 35]. Future prospective studies should be conducted to determine the benefit-risk ratio of early versus delayed SSRF.

Taken together, these findings have direct implications for clinical decision-making in daily practice. They indicate that the maximal treatment effect of SSRF may be in patients a wide age range with moderate to severe overall injury severity and thoracic injury severity contributing significantly to the overall injury severity and therefore support the use of SSRF in this patient cohort. The retrospective and claims-based nature of our data does not allow for the development of a novel clinical pathway without risking over-interpretation. However, the findings are in alignment with current WSES and CWIS consensus statements and guidelines, which the authors support [20, 21].

Some limitations should be acknowledged. One key limitation is derived from the data structure and data availability. As in almost all retrospective observational studies, there is a potential risk for selection bias and unobserved confounders. Furthermore, since this study is based on health claims data, which is designed for billing and administrative purposes and not primarily for medical research, not all variables that would be required for a complete understanding of the effects of SSRF are available in the dataset. For example, factors such as fracture pattern, radiologic imaging and ventilatory parameters as well as outcome measures such as pain and quality-of-life outcomes are not included. Finally, there is likely substantial heterogeneity between the protocols for SSRF and surgeon expertise between different hospitals, which contributes to the observed heterogeneity.

Simultaneously, however, a key strength of our study lies in the data source. Unlike prospective and retrospective clinical studies, which often only include a limited number of study centres, or registry-based studies we analysed real-world data from across all hospital levels in Germany. This provides a more accurate reflection of real-world SSRF utilisation and outcome heterogeneity. Additionally, we used the Elixhauser comorbidity index to comprehensively control for baseline differences.

A distinct methodological feature of our study was the exclusion of patients who died on day 0 or 1 in the conservative group. This was necessary due to a disproportionately high number of very early deaths in that cohort, which likely included patients either deemed non-operable due to severity or managed with palliative intent. By removing these cases, we aimed to reduce confounding by indication and prevent artificial inflation of mortality in the conservative arm. By including all early deaths in the SSRF group but excluding them from the conservative group, our analysis likely underestimates the true survival benefit of surgery. This approach strengthens our findings by avoiding an artificial advantage for SSRF.

Our study also benefited from assessing the injury severity using a validated algorithm that translates ICD-10-GM codes into ISS estimates via ICD-10-CM and AIS mapping, since ISS is not directly coded in administrative data (26). While this process lacks precision on an individual level, prior studies have demonstrated that it performs adequately for population-level stratification, as confirmed by our subgroup analyses (26).

## Conclusion

In conclusion, our results support the increasing evidence suggesting that SSRF improves survival in selected patients with rib fractures, particularly those with moderate injury severity and preserved physiological reserve. The benefit appears to extend to elderly patients. However, longer hospital stays and lack of advantage for early surgery in this cohort highlight the need for individualised decision-making. Future prospective studies should further refine the timing, indication, and perioperative strategies of SSRF.

## Supplementary Information

Below is the link to the electronic supplementary material.

Supplementary Material 1.

Supplementary Material 2.

## Data Availability

The datasets used during the current study are available from the corresponding author on reasonable request where possible from a data protection standpoint.

## Abbreviations

AIS: Abbreviated injury scale
CWIS: Chest wall injury society
HLOS: Hospital length of stay
HR: Hazard ratio
ICD-10 GM: International classification of disease and related health problems,10th Revision, German modification
ICU: Intensive care unit
ISS: Injury severity score
MD: Median difference
OPS: Operations–und Prozedurenschlüssel / Operation and Procedure Code
OR: Odds ratio
PICO: Patients, interventions, controls and outcomes
SSRF: Surgical stabilisation of rib fracuters
WSES: World society of emergency surgery
