# Supplementary material for: The impact of injury severity and age on short-and long-term mortality and hospital length of stay after surgical stabilisation of rib fractures (SSRF): a German population-based propensity-score matched investigation
Source: World J Emerg Surg. 2026 Mar 2;21:21. doi: 10.1186/s13017-026-00682-2 (PMC13059353; doi:10.1186/s13017-026-00682-2)
Supplement: Supplementary file 1 — Supplementary Material 1. [file 13017_2026_682_MOESM1_ESM.pdf]

Supplemental table S1: List of International Classification of Disease, 10th Revision, German Modification (ICD-10-GM) codes used to define the rib fracture cohort, list of ICD-10-GM codes used to define pneumonia and list of procedure codes (Operations- und Prozedurenschlüssel (OPS)) for surgical stabilisation of rib fractures (SSRF).

Code	German name	English translation
ICD-10-GM codes		
S22.41	Rippenserienfraktur mit Beteiligung der ersten Rippe	Serial rib fracture including fracture of the first rib
S22.43	Rippenserienfraktur mit Beteiligung von 3 Rippen	Serial rib fracture affecting 3 ribs
S22.44	Rippenserienfraktur mit Beteiligung von ≥ 4 Rippen	Serial rib fracture affecting ≥ 4 ribs
S22.5	Instabiler Thorax	Unstable thorax
J13	Pneumonie durch Streptococcus pneumoniae	
J14	Pneumonie durch Haemophilus influenzae	Pneumonia caused by Haemophilus influenzae
J15	Pneumonie durch Bakterien, anderorts nicht klassifiziert	Pneumonie caused by bacteria not classified elsewhere
J16	Pneumonie durch sonstige Infektionserreger, anderenorts nicht klassifiziert	Pneumonia caused by other pathogens, not classified elsewhere
J180	Bronchopneumonie, nicht näher bezeichnet	Broncho pneumonia, without further classification
J181	Lobärpneumonie, nicht näher bezeichnet	Lobar pneumonia, without further classification
OPS Codes		
5-346.5		
5-346.c0	Stabilisierung der Thoraxwand, offen chirurgisch, einseitig, mit Reposition und Osteosynthese, 1 Rippe	Surgical stabilisation of the chest wall via open surgery, unilateral, with reduction and osteosynthesis, 1 rib
5-346.c1	Stabilisierung der Thoraxwand, offen chirurgisch, einseitig, mit Reposition und Osteosynthese, 2 Rippen	Surgical stabilisation of the chest wall via open surgery, unilateral, with reduction and osteosynthesis, 2 ribs
5-346.c2	Stabilisierung der Thoraxwand, offen chirurgisch, einseitig, mit Reposition und Osteosynthese, 3-5 Rippen	Surgical stabilisation of the chest wall via open surgery, unilateral, with reduction and osteosynthesis, 3-5 ribs

5-346.c3	Stabilisierung der Thoraxwand, offen chirurgisch, einseitig, mit Reposition und Osteosynthese, ≥ 6 Rippen	Surgical stabilisation of the chest wall via open surgery, unilateral, with reduction and osteosynthesis, ≥ 6 ribs
5-346.d0	Stabilisierung der Thoraxwand, offen chirurgisch, beidseitig, mit Reposition und Osteosynthese, 2 Rippen	Surgical stabilisation of the chest wall via open surgery, bilateral, with reduction and osteosynthesis, 2 ribs
5-346.d1	Stabilisierung der Thoraxwand, offen chirurgisch, beidseitig, mit Reposition und Osteosynthese, 3-4 Rippen	Surgical stabilisation of the chest wall via open surgery, bilateral, with reduction and osteosynthesis, 3-4 ribs
5-346.d2	Stabilisierung der Thoraxwand, offen chirurgisch, beidseitig, mit Reposition und Osteosynthese, 5-8 Rippen	Surgical stabilisation of the chest wall via open surgery, bilateral, with reduction and osteosynthesis, 5-8 ribs
5-346.d3	Stabilisierung der Thoraxwand, offen chirurgisch, beidseitig, mit Reposition und Osteosynthese, ≥ 9 Rippen	Surgical stabilisation of the chest wall via open surgery, bilateral, with reduction and osteosynthesis, ≥ 9 ribs

Supplemental table S2: Assignment of International Classification of Disease, 10th Revision, German Modification (ICD-10-GM) codes to the categories of the individual domains of the Elixhauser score

Item	Group	ICD-10-GM	Name of the condition
Congestive Heart Failure	ELX1	I099	Rheumatische Herzkrankheit, nicht näher bezeichnet
Congestive Heart Failure	ELX1	I110	Hypertensive Herzkrankheit mit (kongestiver) Herzinsuffizienz
Congestive Heart Failure	ELX1	I130	Hypertensive Herz- und Nierenkrankheit mit (kongestiver) Herzinsuffizienz
Congestive Heart Failure	ELX1	I132	Hypertensive Herz- und Nierenkrankheit mit (kongestiver) Herzinsuffizienz und Niereninsuffizienz
Congestive Heart Failure	ELX1	I255	Ischämische Kardiomyopathie
Congestive Heart Failure	ELX1	I420	Dilatative Kardiomyopathie
Congestive Heart Failure	ELX1	I425	Sonstige restriktive Kardiomyopathie
Congestive Heart Failure	ELX1	I426	Alkoholische Kardiomyopathie
Congestive Heart Failure	ELX1	I427	Kardiomyopathie durch Arzneimittel oder sonstige exogene Substanzen
Congestive Heart Failure	ELX1	I428	Sonstige Kardiomyopathien
Congestive Heart Failure	ELX1	I429	Kardiomyopathie, nicht näher bezeichnet
Congestive Heart Failure	ELX1	I43	Kardiomyopathie bei anderenorts klassifizierten Krankheiten
Congestive Heart Failure	ELX1	I50	Herzinsuffizienz
Congestive Heart Failure	ELX1	P290	Herzinsuffizienz beim Neugeborenen
Diabetes Uncomplicated	ELX10	E100	Diabetes mellitus, Typ 1: Mit Koma
Diabetes Uncomplicated	ELX10	E101	Diabetes mellitus, Typ 1: Mit Ketoazidose
Diabetes Uncomplicated	ELX10	E109	Diabetes mellitus, Typ 1: Ohne Komplikationen
Diabetes Uncomplicated	ELX10	E110	Diabetes mellitus, Typ 2: Mit Koma
Diabetes Uncomplicated	ELX10	E111	Diabetes mellitus, Typ 2: Mit Ketoazidose
Diabetes Uncomplicated	ELX10	E119	Diabetes mellitus, Typ 2: Ohne Komplikationen
Diabetes Uncomplicated	ELX10	E120	Diabetes mellitus in Verbindung mit Fehl- oder Mangelernährung [Malnutrition]: Mit Koma
Diabetes Uncomplicated	ELX10	E121	Diabetes mellitus in Verbindung mit Fehl- oder Mangelernährung [Malnutrition]: Mit Ketoazidose
Diabetes Uncomplicated	ELX10	E129	Diabetes mellitus in Verbindung mit Fehl- oder Mangelernährung [Malnutrition]: Ohne Komplikationen
Diabetes Uncomplicated	ELX10	E130	Sonstiger näher bezeichneter Diabetes mellitus: Mit Koma
Diabetes Uncomplicated	ELX10	E131	Sonstiger näher bezeichneter Diabetes mellitus: Mit Ketoazidose
Diabetes Uncomplicated	ELX10	E139	Sonstiger näher bezeichneter Diabetes mellitus: Ohne Komplikationen
Diabetes Uncomplicated	ELX10	E140	Nicht näher bezeichneter Diabetes mellitus: Mit Koma

Diabetes Uncomplicated	ELX10	E141	Nicht näher bezeichneter Diabetes mellitus: Mit Ketoazidose
Diabetes Uncomplicated	ELX10	E149	Nicht näher bezeichneter Diabetes mellitus: Ohne Komplikationen
Diabetes Complicated	ELX11	E102	Diabetes mellitus, Typ 1: Mit Nierenkomplikationen
Diabetes Complicated	ELX11	E103	Diabetes mellitus, Typ 1: Mit Augenkomplikationen
Diabetes Complicated	ELX11	E104	Diabetes mellitus, Typ 1: Mit neurologischen Komplikationen
Diabetes Complicated	ELX11	E105	Diabetes mellitus, Typ 1: Mit peripheren vaskulären Komplikationen
Diabetes Complicated	ELX11	E106	Diabetes mellitus, Typ 1: Mit sonstigen näher bezeichneten Komplikationen
Diabetes Complicated	ELX11	E107	Diabetes mellitus, Typ 1: Mit multiplen Komplikationen
Diabetes Complicated	ELX11	E108	Diabetes mellitus, Typ 1: Mit nicht näher bezeichneten Komplikationen
Diabetes Complicated	ELX11	E112	Diabetes mellitus, Typ 2: Mit Nierenkomplikationen
Diabetes Complicated	ELX11	E113	Diabetes mellitus, Typ 2: Mit Augenkomplikationen
Diabetes Complicated	ELX11	E114	Diabetes mellitus, Typ 2: Mit neurologischen Komplikationen
Diabetes Complicated	ELX11	E115	Diabetes mellitus, Typ 2: Mit peripheren vaskulären Komplikationen
Diabetes Complicated	ELX11	E116	Diabetes mellitus, Typ 2: Mit sonstigen näher bezeichneten Komplikationen
Diabetes Complicated	ELX11	E117	Diabetes mellitus, Typ 2: Mit multiplen Komplikationen
Diabetes Complicated	ELX11	E118	Diabetes mellitus, Typ 2: Mit nicht näher bezeichneten Komplikationen
Diabetes Complicated	ELX11	E122	Diabetes mellitus in Verbindung mit Fehl- oder Mangelernährung [Malnutrition]: Mit Nierenkomplikationen
Diabetes Complicated	ELX11	E123	Diabetes mellitus in Verbindung mit Fehl- oder Mangelernährung [Malnutrition]: Mit Augenkomplikationen
Diabetes Complicated	ELX11	E124	Diabetes mellitus in Verbindung mit Fehl- oder Mangelernährung [Malnutrition]: Mit neurologischen Komplikationen
Diabetes Complicated	ELX11	E125	Diabetes mellitus in Verbindung mit Fehl- oder Mangelernährung [Malnutrition]: Mit peripheren vaskulären Komplikationen
Diabetes Complicated	ELX11	E126	Diabetes mellitus in Verbindung mit Fehl- oder Mangelernährung [Malnutrition]: Mit sonstigen näher bezeichneten Komplikationen
Diabetes Complicated	ELX11	E127	Diabetes mellitus in Verbindung mit Fehl- oder Mangelernährung [Malnutrition]: Mit multiplen Komplikationen
Diabetes Complicated	ELX11	E128	Diabetes mellitus in Verbindung mit Fehl- oder Mangelernährung [Malnutrition]: Mit nicht näher bezeichneten Komplikationen
Diabetes Complicated	ELX11	E132	Sonstiger näher bezeichneter Diabetes mellitus: Mit Nierenkomplikationen
Diabetes Complicated	ELX11	E133	Sonstiger näher bezeichneter Diabetes mellitus: Mit Augenkomplikationen
Diabetes Complicated	ELX11	E134	Sonstiger näher bezeichneter Diabetes mellitus: Mit neurologischen Komplikationen
Diabetes Complicated	ELX11	E135	Sonstiger näher bezeichneter Diabetes mellitus: Mit peripheren vaskulären Komplikationen
Diabetes Complicated	ELX11	E136	Sonstiger näher bezeichneter Diabetes mellitus: Mit sonstigen näher bezeichneten Komplikationen

Diabetes Complicated	ELX11	E137	Sonstiger näher bezeichneter Diabetes mellitus: Mit multiplen Komplikationen
Diabetes Complicated	ELX11	E138	Sonstiger näher bezeichneter Diabetes mellitus: Mit nicht näher bezeichneten Komplikationen
Diabetes Complicated	ELX11	E142	Nicht näher bezeichneter Diabetes mellitus: Mit Nierenkomplikationen
Diabetes Complicated	ELX11	E143	Nicht näher bezeichneter Diabetes mellitus: Mit Augenkomplikationen
Diabetes Complicated	ELX11	E144	Nicht näher bezeichneter Diabetes mellitus: Mit neurologischen Komplikationen
Diabetes Complicated	ELX11	E145	Nicht näher bezeichneter Diabetes mellitus: Mit peripheren vaskulären Komplikationen
Diabetes Complicated	ELX11	E146	Nicht näher bezeichneter Diabetes mellitus: Mit sonstigen näher bezeichneten Komplikationen
Diabetes Complicated	ELX11	E147	Nicht näher bezeichneter Diabetes mellitus: Mit multiplen Komplikationen
Diabetes Complicated	ELX11	E148	Nicht näher bezeichneter Diabetes mellitus: Mit nicht näher bezeichneten Komplikationen
Hypothyroidism	ELX12	E00	Angeborenes Jodmangelsyndrom
Hypothyroidism	ELX12	E01	Jodmangelbedingte Schilddrüsenkrankheiten und verwandte Zustände
Hypothyroidism	ELX12	E02	Subklinische Jodmangel-Hypothyreose
Hypothyroidism	ELX12	E03	Sonstige Hypothyreose
Hypothyroidism	ELX12	E890	Hypothyreose nach medizinischen Maßnahmen
Renal Failure	ELX13	I120	Hypertensive Nierenkrankheit mit Niereninsuffizienz
Renal Failure	ELX13	I131	Hypertensive Herz- und Nierenkrankheit mit Niereninsuffizienz
Renal Failure	ELX13	N18	Chronische Nierenkrankheit
Renal Failure	ELX13	N19	Nicht näher bezeichnete Niereninsuffizienz
Renal Failure	ELX13	N250	Renale Osteodystrophie
Renal Failure	ELX13	Z490	Vorbereitung auf die Dialyse
Renal Failure	ELX13	Z491	Extrakorporale Dialyse
Renal Failure	ELX13	Z492	Sonstige Dialyse
Renal Failure	ELX13	Z940	Zustand nach Nierentransplantation
Renal Failure	ELX13	Z992	Langzeitige Abhängigkeit von Dialyse bei Niereninsuffizienz
Liver Disease	ELX14	B18	Chronische Virushepatitis
Liver Disease	ELX14	I85	Ösophagusvarizen
Liver Disease	ELX14	I864	Magenvarizen
Liver Disease	ELX14	I982	Ösophagus- und Magenvarizen bei anderenorts klassifizierten Krankheiten, ohne Angabe einer Blutung
Liver Disease	ELX14	K70	Alkoholische Leberkrankheit
Liver Disease	ELX14	K711	Toxische Leberkrankheit mit Lebernekrose
Liver Disease	ELX14	K713	Toxische Leberkrankheit mit chronisch-persistierender Hepatitis
Liver Disease	ELX14	K714	Toxische Leberkrankheit mit chronischer lobulärer Hepatitis
Liver Disease	ELX14	K715	Toxische Leberkrankheit mit chronisch-aktiver Hepatitis
Liver Disease	ELX14	K717	Toxische Leberkrankheit mit Fibrose und Zirrhose der Leber
Liver Disease	ELX14	K72	Leberversagen, anderenorts nicht klassifiziert
Liver Disease	ELX14	K73	Chronische Hepatitis, anderenorts nicht klassifiziert
Liver Disease	ELX14	K74	Fibrose und Zirrhose der Leber
Liver Disease	ELX14	K760	Fettleber [fettige Degeneration], anderenorts nicht klassifiziert
Liver Disease	ELX14	K762	Zentrale hämorrhagische Lebernekrose

Liver Disease	ELX14	K763	Leberinfarkt
Liver Disease	ELX14	K764	Peliosis hepatis
Liver Disease	ELX14	K765	Venöse okklusive Leberkrankheit [Stuart-Bras-Syndrom]
Liver Disease	ELX14	K766	Portale Hypertonie
Liver Disease	ELX14	K767	Hepatorenales Syndrom
Liver Disease	ELX14	K768	Sonstige näher bezeichnete Krankheiten der Leber
Liver Disease	ELX14	K769	Leberkrankheit, nicht näher bezeichnet
Liver Disease	ELX14	Z944	Zustand nach Lebertransplantation
Peptic Ulcer Disease excluding bleeding	ELX15	K257	Ulcus ventriculi: Chronisch, ohne Blutung oder Perforation
Peptic Ulcer Disease excluding bleeding	ELX15	K259	Ulcus ventriculi: Weder als akut noch als chronisch bezeichnet, ohne Blutung oder Perforation
Peptic Ulcer Disease excluding bleeding	ELX15	K267	Ulcus duodeni: Chronisch, ohne Blutung oder Perforation
Peptic Ulcer Disease excluding bleeding	ELX15	K269	Ulcus duodeni: Weder als akut noch als chronisch bezeichnet, ohne Blutung oder Perforation
Peptic Ulcer Disease excluding bleeding	ELX15	K277	Ulcus pepticum, Lokalisation nicht näher bezeichnet: Chronisch, ohne Blutung oder Perforation
Peptic Ulcer Disease excluding bleeding	ELX15	K279	Ulcus pepticum, Lokalisation nicht näher bezeichnet: Weder als akut noch als chronisch bezeichnet, ohne Blutung oder Perforation
Peptic Ulcer Disease excluding bleeding	ELX15	K287	Ulcus pepticum jejuni: Chronisch, ohne Blutung oder Perforation
Peptic Ulcer Disease excluding bleeding	ELX15	K289	Ulcus pepticum jejuni: Weder als akut noch als chronisch bezeichnet, ohne Blutung oder Perforation
AIDS/HIV	ELX16	B20	Infektiöse und parasitäre Krankheiten infolge HIV-Krankheit [Humane Immundefizienz-Viruskrankheit]
AIDS/HIV	ELX16	B21	Bösartige Neubildungen infolge HIV-Krankheit [Humane Immundefizienz-Viruskrankheit]
AIDS/HIV	ELX16	B22	Sonstige näher bezeichnete Krankheiten infolge HIV-Krankheit [Humane Immundefizienz-Viruskrankheit]
AIDS/HIV	ELX16	B24	Nicht näher bezeichnete HIV-Krankheit [Humane Immundefizienz-Viruskrankheit]
Lymphoma	ELX17	C81	Hodgkin-Lymphom [Lymphogranulomatose]
Lymphoma	ELX17	C82	Follikuläres Lymphom
Lymphoma	ELX17	C83	Nicht follikuläres Lymphom
Lymphoma	ELX17	C84	Reifzellige T/NK-Zell-Lymphome
Lymphoma	ELX17	C85	Sonstige und nicht näher bezeichnete Typen des Non-Hodgkin-Lymphoms
Lymphoma	ELX17	C88	Bösartige immunproliferative Krankheiten
Lymphoma	ELX17	C900	Multiples Myelom
Lymphoma	ELX17	C902	Extramedulläres Plasmozytom
Lymphoma	ELX17	C96	Sonstige und nicht näher bezeichnete bösartige Neubildungen des lymphatischen, blutbildenden und verwandten Gewebes
Metastatic Cancer	ELX18	C77	Sekundäre und nicht näher bezeichnete bösartige Neubildung der Lymphknoten
Metastatic Cancer	ELX18	C78	Sekundäre bösartige Neubildung der Atmungs- und Verdauungsorgane
Metastatic Cancer	ELX18	C79	Sekundäre bösartige Neubildung an sonstigen und nicht näher bezeichneten Lokalisationen
Metastatic Cancer	ELX18	C80	Bösartige Neubildung ohne Angabe der Lokalisation
Solid Tumor without Metastasis	ELX19	C00	Bösartige Neubildung der Lippe

Solid Tumor without Metastasis	ELX19	C01	Bösartige Neubildung des Zungengrundes
Solid Tumor without Metastasis	ELX19	C02	Bösartige Neubildung sonstiger und nicht näher bezeichneter Teile der Zunge
Solid Tumor without Metastasis	ELX19	C03	Bösartige Neubildung des Zahnfleisches
Solid Tumor without Metastasis	ELX19	C04	Bösartige Neubildung des Mundbodens
Solid Tumor without Metastasis	ELX19	C05	Bösartige Neubildung des Gaumens
Solid Tumor without Metastasis	ELX19	C06	Bösartige Neubildung sonstiger und nicht näher bezeichneter Teile des Mundes
Solid Tumor without Metastasis	ELX19	C07	Bösartige Neubildung der Parotis
Solid Tumor without Metastasis	ELX19	C08	Bösartige Neubildung sonstiger und nicht näher bezeichneter großer Speicheldrüsen
Solid Tumor without Metastasis	ELX19	C09	Bösartige Neubildung der Tonsille
Solid Tumor without Metastasis	ELX19	C10	Bösartige Neubildung des Oropharynx
Solid Tumor without Metastasis	ELX19	C11	Bösartige Neubildung des Nasopharynx
Solid Tumor without Metastasis	ELX19	C12	Bösartige Neubildung des Recessus piriformis
Solid Tumor without Metastasis	ELX19	C13	Bösartige Neubildung des Hypopharynx
Solid Tumor without Metastasis	ELX19	C14	Bösartige Neubildung sonstiger und ungenau bezeichneter Lokalisationen der Lippe, der Mundhöhle und des Pharynx
Solid Tumor without Metastasis	ELX19	C15	Bösartige Neubildung des Ösophagus
Solid Tumor without Metastasis	ELX19	C16	Bösartige Neubildung des Magens
Solid Tumor without Metastasis	ELX19	C17	Bösartige Neubildung des Dünndarmes
Solid Tumor without Metastasis	ELX19	C18	Bösartige Neubildung des Kolons
Solid Tumor without Metastasis	ELX19	C19	Bösartige Neubildung am Rektosigmoid, Übergang
Solid Tumor without Metastasis	ELX19	C20	Bösartige Neubildung des Rektums
Solid Tumor without Metastasis	ELX19	C21	Bösartige Neubildung des Anus und des Analkanals
Solid Tumor without Metastasis	ELX19	C22	Bösartige Neubildung der Leber und der intrahepatischen Gallengänge
Solid Tumor without Metastasis	ELX19	C23	Bösartige Neubildung der Gallenblase
Solid Tumor without Metastasis	ELX19	C24	Bösartige Neubildung sonstiger und nicht näher bezeichneter Teile der Gallenwege
Solid Tumor without Metastasis	ELX19	C25	Bösartige Neubildung des Pankreas
Solid Tumor without Metastasis	ELX19	C26	Bösartige Neubildung sonstiger und ungenau bezeichneter Verdauungsorgane
Solid Tumor without Metastasis	ELX19	C30	Bösartige Neubildung der Nasenhöhle und des Mittelohres
Solid Tumor without Metastasis	ELX19	C31	Bösartige Neubildung der Nasennebenhöhlen
Solid Tumor without Metastasis	ELX19	C32	Bösartige Neubildung des Larynx

Solid Tumor without Metastasis	ELX19	C33	Bösartige Neubildung der Trachea
Solid Tumor without Metastasis	ELX19	C34	Bösartige Neubildung der Bronchien und der Lunge
Solid Tumor without Metastasis	ELX19	C37	Bösartige Neubildung des Thymus
Solid Tumor without Metastasis	ELX19	C38	Bösartige Neubildung des Herzens, des Mediastinums und der Pleura
Solid Tumor without Metastasis	ELX19	C39	Bösartige Neubildung sonstiger und ungenau bezeichneter Lokalisationen des Atmungssystems und sonstiger intrathorakaler Organe
Solid Tumor without Metastasis	ELX19	C40	Bösartige Neubildung des Knochens und des Gelenkknorpels der Extremitäten
Solid Tumor without Metastasis	ELX19	C41	Bösartige Neubildung des Knochens und des Gelenkknorpels sonstiger und nicht näher bezeichneter Lokalisationen
Solid Tumor without Metastasis	ELX19	C43	Bösartiges Melanom der Haut
Solid Tumor without Metastasis	ELX19	C45	Mesotheliom
Solid Tumor without Metastasis	ELX19	C46	Kaposi-Sarkom [Sarcoma idiopathicum multiplex haemorrhagicum]
Solid Tumor without Metastasis	ELX19	C47	Bösartige Neubildung der peripheren Nerven und des autonomen Nervensystems
Solid Tumor without Metastasis	ELX19	C48	Bösartige Neubildung des Retroperitoneums und des Peritoneums
Solid Tumor without Metastasis	ELX19	C49	Bösartige Neubildung sonstigen Bindegewebes und anderer Weichteilgewebe
Solid Tumor without Metastasis	ELX19	C50	Bösartige Neubildung der Brustdrüse [Mamma]
Solid Tumor without Metastasis	ELX19	C51	Bösartige Neubildung der Vulva
Solid Tumor without Metastasis	ELX19	C52	Bösartige Neubildung der Vagina
Solid Tumor without Metastasis	ELX19	C53	Bösartige Neubildung der Cervix uteri
Solid Tumor without Metastasis	ELX19	C54	Bösartige Neubildung des Corpus uteri
Solid Tumor without Metastasis	ELX19	C55	Bösartige Neubildung des Uterus, Teil nicht näher bezeichnet
Solid Tumor without Metastasis	ELX19	C56	Bösartige Neubildung des Ovars
Solid Tumor without Metastasis	ELX19	C57	Bösartige Neubildung sonstiger und nicht näher bezeichneter weiblicher Genitalorgane
Solid Tumor without Metastasis	ELX19	C58	Bösartige Neubildung der Plazenta
Solid Tumor without Metastasis	ELX19	C60	Bösartige Neubildung des Penis
Solid Tumor without Metastasis	ELX19	C61	Bösartige Neubildung der Prostata
Solid Tumor without Metastasis	ELX19	C62	Bösartige Neubildung des Hodens
Solid Tumor without Metastasis	ELX19	C63	Bösartige Neubildung sonstiger und nicht näher bezeichneter männlicher Genitalorgane
Solid Tumor without Metastasis	ELX19	C64	Bösartige Neubildung der Niere, ausgenommen Nierenbecken
Solid Tumor without Metastasis	ELX19	C65	Bösartige Neubildung des Nierenbeckens
Solid Tumor without Metastasis	ELX19	C66	Bösartige Neubildung des Ureters

Solid Tumor without Metastasis	ELX19	C67	Bösartige Neubildung der Harnblase
Solid Tumor without Metastasis	ELX19	C68	Bösartige Neubildung sonstiger und nicht näher bezeichneter Harnorgane
Solid Tumor without Metastasis	ELX19	C69	Bösartige Neubildung des Auges und der Augenanhangsgebilde
Solid Tumor without Metastasis	ELX19	C70	Bösartige Neubildung der Meningen
Solid Tumor without Metastasis	ELX19	C71	Bösartige Neubildung des Gehirns
Solid Tumor without Metastasis	ELX19	C72	Bösartige Neubildung des Rückenmarkes, der Hirnnerven und anderer Teile des Zentralnervensystems
Solid Tumor without Metastasis	ELX19	C73	Bösartige Neubildung der Schilddrüse
Solid Tumor without Metastasis	ELX19	C74	Bösartige Neubildung der Nebenniere
Solid Tumor without Metastasis	ELX19	C75	Bösartige Neubildung sonstiger endokriner Drüsen und verwandter Strukturen
Solid Tumor without Metastasis	ELX19	C76	Bösartige Neubildung sonstiger und ungenau bezeichneter Lokalisationen
Solid Tumor without Metastasis	ELX19	C97	Bösartige Neubildungen als Primärtumoren an mehreren Lokalisationen
Caridiac Arrhythmia	ELX2	I441	Atrioventrikulärer Block 2. Grades
Caridiac Arrhythmia	ELX2	I442	Atrioventrikulärer Block 3. Grades
Caridiac Arrhythmia	ELX2	I443	Sonstiger und nicht näher bezeichneter atrioventrikulärer Block
Caridiac Arrhythmia	ELX2	I456	Präexzitations-Syndrom
Caridiac Arrhythmia	ELX2	I459	Kardiale Erregungsleitungsstörung, nicht näher bezeichnet
Caridiac Arrhythmia	ELX2	I47	Paroxysmale Tachykardie
Caridiac Arrhythmia	ELX2	I48	Vorhofflimmern und Vorhofflattern
Caridiac Arrhythmia	ELX2	I49	Sonstige kardiale Arrhythmien
Caridiac Arrhythmia	ELX2	R000	Tachykardie, nicht näher bezeichnet
Caridiac Arrhythmia	ELX2	R001	Bradykardie, nicht näher bezeichnet
Caridiac Arrhythmia	ELX2	R008	Sonstige und nicht näher bezeichnete Störungen des Herzschlages
Caridiac Arrhythmia	ELX2	T821	Mechanische Komplikation durch ein kardiales elektronisches Gerät
Caridiac Arrhythmia	ELX2	Z450	Anpassung und Handhabung eines kardialen (elektronischen) Geräts
Caridiac Arrhythmia	ELX2	Z950	Vorhandensein eines kardialen elektronischen Geräts
Rheumatoid Arthsitis/collagen	ELX20	L940	Sclerodermia circumscripta [Morphaea]
Rheumatoid Arthsitis/collagen	ELX20	L941	Lineare oder bandförmige Sklerodermie
Rheumatoid Arthsitis/collagen	ELX20	L943	Sklerodaktylie
Rheumatoid Arthsitis/collagen	ELX20	M05	Seropositive chronische Polyarthritis
Rheumatoid Arthsitis/collagen	ELX20	M06	Sonstige chronische Polyarthritis
Rheumatoid Arthsitis/collagen	ELX20	M08	Juvenile Arthritis
Rheumatoid Arthsitis/collagen	ELX20	M120	Chronische postrheumatische Arthritis [Jaccoud-Arthritis]
Rheumatoid Arthsitis/collagen	ELX20	M123	Palindromer Rheumatismus

Rheumatoid Arthsitis/collagen	ELX20	M30	Panarteriitis nodosa und verwandte Zustände
Rheumatoid Arthsitis/collagen	ELX20	M310	Hypersensitivitätsangiitis
Rheumatoid Arthsitis/collagen	ELX20	M311	Thrombotische Mikroangiopathie
Rheumatoid Arthsitis/collagen	ELX20	M312	Letales Mittelliniengranulom
Rheumatoid Arthsitis/collagen	ELX20	M313	Wegener-Granulomatose
Rheumatoid Arthsitis/collagen	ELX20	M32	Systemischer Lupus erythematodes
Rheumatoid Arthsitis/collagen	ELX20	M33	Dermatomyositis-Polymyositis
Rheumatoid Arthsitis/collagen	ELX20	M34	Systemische Sklerose
Rheumatoid Arthsitis/collagen	ELX20	M35	Sonstige Krankheiten mit Systembeteiligung des Bindegewebes
Rheumatoid Arthsitis/collagen	ELX20	M45	Spondylitis ankylosans
Rheumatoid Arthsitis/collagen	ELX20	M461	Sakroiliitis, anderenorts nicht klassifiziert
Rheumatoid Arthsitis/collagen	ELX20	M468	Sonstige näher bezeichnete entzündliche Spondylopathien
Rheumatoid Arthsitis/collagen	ELX20	M469	Entzündliche Spondylopathie, nicht näher bezeichnet
Coagulopathy	ELX21	D65	Disseminierte intravasale Gerinnung [Defibrinationssyndrom]
Coagulopathy	ELX21	D66	Hereditärer Faktor-VIII-Mangel
Coagulopathy	ELX21	D67	Hereditärer Faktor-IX-Mangel
Coagulopathy	ELX21	D68	Sonstige Koagulopathien
Coagulopathy	ELX21	D691	Qualitative Thrombozytendefekte
Coagulopathy	ELX21	D693	Idiopathische thrombozytopenische Purpura
Coagulopathy	ELX21	D694	Sonstige primäre Thrombozytopenie
Coagulopathy	ELX21	D695	Sekundäre Thrombozytopenie
Coagulopathy	ELX21	D696	Thrombozytopenie, nicht näher bezeichnet
Obesity	ELX22	E66	Adipositas
Weight Loss	ELX23	E40	Kwashiorkor
Weight Loss	ELX23	E41	Alimentärer Marasmus
Weight Loss	ELX23	E42	Kwashiorkor-Marasmus
Weight Loss	ELX23	E43	Nicht näher bezeichnete erhebliche Energie- und Eiweißmangelernährung
Weight Loss	ELX23	E44	Energie- und Eiweißmangelernährung mäßigen und leichten Grades
Weight Loss	ELX23	E45	Entwicklungsverzögerung durch Energie- und Eiweißmangelernährung
Weight Loss	ELX23	E46	Nicht näher bezeichnete Energie- und Eiweißmangelernährung
Weight Loss	ELX23	R634	Abnorme Gewichtsabnahme
Weight Loss	ELX23	R64	Kachexie
Fluid and Ecletrolyte Disorders	ELX24	E222	Syndrom der inadäquaten Sekretion von Adiuretin
Fluid and Ecletrolyte Disorders	ELX24	E86	Volumenmangel
Fluid and Ecletrolyte Disorders	ELX24	E87	Sonstige Störungen des Wasser- und Elektrolythaushaltes sowie des Säure-Basen-Gleichgewichts
Blood Loss Anemia	ELX25	D500	Eisenmangelanämie nach Blutverlust (chronisch)

Deficiency Anemia	ELX26	D508	Sonstige Eisenmangelanämien
Deficiency Anemia	ELX26	D509	Eisenmangelanämie, nicht näher bezeichnet
Deficiency Anemia	ELX26	D51	Vitamin-B12-Mangelanämie
Deficiency Anemia	ELX26	D52	Folsäure-Mangelanämie
Deficiency Anemia	ELX26	D53	Sonstige alimentäre Anämien
Alcohol Abuse	ELX27	E52	Niazinmangel [Pellagra]
Alcohol Abuse	ELX27	F10	Psychische und Verhaltensstörungen durch Alkohol
Alcohol Abuse	ELX27	G621	Alkohol-Polyneuropathie
Alcohol Abuse	ELX27	I426	Alkoholische Kardiomyopathie
Alcohol Abuse	ELX27	K292	Alkoholgastritis
Alcohol Abuse	ELX27	K700	Alkoholische Fettleber
Alcohol Abuse	ELX27	K703	Alkoholische Leberzirrhose
Alcohol Abuse	ELX27	K709	Alkoholische Leberkrankheit, nicht näher bezeichnet
Alcohol Abuse	ELX27	T51	Toxische Wirkung von Alkohol
Alcohol Abuse	ELX27	Z502	Rehabilitationsmaßnahmen bei Alkoholismus
Drug Abuse	ELX28	F11	Psychische und Verhaltensstörungen durch Opioide
Drug Abuse	ELX28	F12	Psychische und Verhaltensstörungen durch Cannabinoide
Drug Abuse	ELX28	F13	Psychische und Verhaltensstörungen durch Sedativa oder Hypnotika
Drug Abuse	ELX28	F14	Psychische und Verhaltensstörungen durch Kokain
Drug Abuse	ELX28	F15	Psychische und Verhaltensstörungen durch andere Stimulanzien, einschließlich Koffein
Drug Abuse	ELX28	F16	Psychische und Verhaltensstörungen durch Halluzinogene
Drug Abuse	ELX28	F18	Psychische und Verhaltensstörungen durch flüchtige Lösungsmittel
Drug Abuse	ELX28	F19	Psychische und Verhaltensstörungen durch multiplen Substanzgebrauch und Konsum anderer psychotroper Substanzen
Psychoses	ELX29	F20	Schizophrenie
Psychoses	ELX29	F22	Anhaltende wahnhafte Störungen
Psychoses	ELX29	F23	Akute vorübergehende psychotische Störungen
Psychoses	ELX29	F24	Induzierte wahnhafte Störung
Psychoses	ELX29	F25	Schizoaffektive Störungen
Psychoses	ELX29	F28	Sonstige nichtorganische psychotische Störungen
Psychoses	ELX29	F29	Nicht näher bezeichnete nichtorganische Psychose
Psychoses	ELX29	F302	Manie mit psychotischen Symptomen
Psychoses	ELX29	F312	Bipolare affektive Störung, gegenwärtig manische Episode mit psychotischen Symptomen
Psychoses	ELX29	F315	Bipolare affektive Psychose, gegenwärtig schwere depressive Episode mit psychotischen Symptomen
Valvular Disease	ELX3	A520	Kardiovaskuläre Syphilis
Valvular Disease	ELX3	I05	Rheumatische Mitralklappenkrankheiten
Valvular Disease	ELX3	I06	Rheumatische Aortenklappenkrankheiten
Valvular Disease	ELX3	I07	Rheumatische Trikuspidalklappenkrankheiten
Valvular Disease	ELX3	I08	Krankheiten mehrerer Herzklappen
Valvular Disease	ELX3	I091	Rheumatische Krankheiten des Endokards, Herzklappe nicht näher bezeichnet
Valvular Disease	ELX3	I098	Sonstige näher bezeichnete rheumatische Herzkrankheiten
Valvular Disease	ELX3	I34	Nichtrheumatische Mitralklappenkrankheiten
Valvular Disease	ELX3	I35	Nichtrheumatische Aortenklappenkrankheiten
Valvular Disease	ELX3	I36	Nichtrheumatische Trikuspidalklappenkrankheiten
Valvular Disease	ELX3	I37	Pulmonalklappenkrankheiten
Valvular Disease	ELX3	I38	Endokarditis, Herzklappe nicht näher bezeichnet

Valvular Disease	ELX3	I39	Endokarditis und Herzklappenkrankheiten bei anderenorts klassifizierten Krankheiten
Valvular Disease	ELX3	Q230	Angeborene Aortenklappenstenose
Valvular Disease	ELX3	Q231	Angeborene Aortenklappeninsuffizienz
Valvular Disease	ELX3	Q232	Angeborene Mitralklappenstenose
Valvular Disease	ELX3	Q233	Angeborene Mitralklappeninsuffizienz
Valvular Disease	ELX3	Z952	Vorhandensein einer künstlichen Herzklappe
Valvular Disease	ELX3	Z953	Vorhandensein einer xenogenen Herzklappe
Valvular Disease	ELX3	Z954	Vorhandensein eines anderen Herzklappenersatzes
Depression	ELX30	F204	Postschizophrene Depression
Depression	ELX30	F313	Bipolare affektive Störung, gegenwärtig leichte oder mittelgradige depressive Episode
Depression	ELX30	F314	Bipolare affektive Störung, gegenwärtig schwere depressive Episode ohne psychotische Symptome
Depression	ELX30	F315	Bipolare affektive Psychose, gegenwärtig schwere depressive Episode mit psychotischen Symptomen
Depression	ELX30	F32	Depressive Episode
Depression	ELX30	F33	Rezidivierende depressive Störung
Depression	ELX30	F341	Dysthymia
Depression	ELX30	F412	Angst und depressive Störung, gemischt
Depression	ELX30	F432	Anpassungsstörungen
Pulmonary Circulation Disorders	ELX4	I26	Lungenembolie
Pulmonary Circulation Disorders	ELX4	I27	Sonstige pulmonale Herzkrankheiten
Pulmonary Circulation Disorders	ELX4	I280	Arteriovenöse Fistel der Lungengefäße
Pulmonary Circulation Disorders	ELX4	I288	Sonstige näher bezeichnete Krankheiten der Lungengefäße
Pulmonary Circulation Disorders	ELX4	I289	Krankheit der Lungengefäße, nicht näher bezeichnet
Peripheral Vascular Disorders	ELX5	I70	Atherosklerose
Peripheral Vascular Disorders	ELX5	I71	Aortenaneurysma und -dissektion
Peripheral Vascular Disorders	ELX5	I731	Thrombangiitis obliterans [Endangiitis von-Winiwarter-Buerger]
Peripheral Vascular Disorders	ELX5	I738	Sonstige näher bezeichnete periphere Gefäßkrankheiten
Peripheral Vascular Disorders	ELX5	I739	Periphere Gefäßkrankheit, nicht näher bezeichnet
Peripheral Vascular Disorders	ELX5	I771	Arterienstriktur
Peripheral Vascular Disorders	ELX5	I790	Aortenaneurysma bei anderenorts klassifizierten Krankheiten
Peripheral Vascular Disorders	ELX5	I792	Periphere Angiopathie bei anderenorts klassifizierten Krankheiten
Peripheral Vascular Disorders	ELX5	K551	Chronische Gefäßkrankheiten des Darmes
Peripheral Vascular Disorders	ELX5	K558	Sonstige Gefäßkrankheiten des Darmes
Peripheral Vascular Disorders	ELX5	K559	Gefäßkrankheit des Darmes, nicht näher bezeichnet
Peripheral Vascular Disorders	ELX5	Z958	Vorhandensein von sonstigen kardialen oder vaskulären Implantaten oder Transplantaten
Peripheral Vascular Disorders	ELX5	Z959	Vorhandensein von kardialem oder vaskulärem Implantat oder Transplantat, nicht näher bezeichnet
Hypertension	ELX6	I10	Essentielle (primäre) Hypertonie

Hypertension	ELX6	I11	Hypertensive Herzkrankheit
Hypertension	ELX6	I12	Hypertensive Nierenkrankheit
Hypertension	ELX6	I13	Hypertensive Herz- und Nierenkrankheit
Hypertension	ELX6	I15	Sekundäre Hypertonie
Paralysis	ELX7	G041	Tropische spastische Paraplegie
Paralysis	ELX7	G114	Hereditäre spastische Paraplegie
Paralysis	ELX7	G801	Spastische diplegische Zerebralparese
Paralysis	ELX7	G802	Infantile hemiplegische Zerebralparese
Paralysis	ELX7	G81	Hemiparese und Hemiplegie
Paralysis	ELX7	G82	Paraparese und Paraplegie, Tetraparese und Tetraplegie
Paralysis	ELX7	G830	Diparese und Diplegie der oberen Extremitäten
Paralysis	ELX7	G831	Monoparese und Monoplegie einer unteren Extremität
Paralysis	ELX7	G832	Monoparese und Monoplegie einer oberen Extremität
Paralysis	ELX7	G833	Monoparese und Monoplegie, nicht näher bezeichnet
Paralysis	ELX7	G834	Cauda- (equina-) Syndrom
Paralysis	ELX7	G839	Lähmungssyndrom, nicht näher bezeichnet
Other Neurological Disorders	ELX8	G10	Chorea Huntington
Other Neurological Disorders	ELX8	G11	Hereditäre Ataxie
Other Neurological Disorders	ELX8	G12	Spinale Muskelatrophie und verwandte Syndrome
Other Neurological Disorders	ELX8	G13	Systematrophien, vorwiegend das Zentralnervensystem betreffend, bei anderenorts klassifizierten Krankheiten
Other Neurological Disorders	ELX8	G20	Primäres Parkinson-Syndrom
Other Neurological Disorders	ELX8	G21	Sekundäres Parkinson-Syndrom
Other Neurological Disorders	ELX8	G22	Parkinson-Syndrom bei anderenorts klassifizierten Krankheiten
Other Neurological Disorders	ELX8	G254	Arzneimittelinduzierte Chorea
Other Neurological Disorders	ELX8	G255	Sonstige Chorea
Other Neurological Disorders	ELX8	G312	Degeneration des Nervensystems durch Alkohol
Other Neurological Disorders	ELX8	G318	Sonstige näher bezeichnete degenerative Krankheiten des Nervensystems
Other Neurological Disorders	ELX8	G319	Degenerative Krankheit des Nervensystems, nicht näher bezeichnet
Other Neurological Disorders	ELX8	G32	Sonstige degenerative Krankheiten des Nervensystems bei anderenorts klassifizierten Krankheiten
Other Neurological Disorders	ELX8	G35	Multiple Sklerose [Encephalomyelitis disseminata]
Other Neurological Disorders	ELX8	G36	Sonstige akute disseminierte Demyelinisation
Other Neurological Disorders	ELX8	G37	Sonstige demyelinisierende Krankheiten des Zentralnervensystems
Other Neurological Disorders	ELX8	G40	Epilepsie
Other Neurological Disorders	ELX8	G41	Status epilepticus

Other Neurological Disorders	ELX8	G931	Anoxische Hirnschädigung, anderenorts nicht klassifiziert
Other Neurological Disorders	ELX8	G934	Enzephalopathie, nicht näher bezeichnet
Other Neurological Disorders	ELX8	R470	Dysphasie und Aphasie
Other Neurological Disorders	ELX8	R56	Krämpfe, anderenorts nicht klassifiziert
Chronic Pulmonary Disease	ELX9	I278	Sonstige näher bezeichnete pulmonale Herzkrankheiten
Chronic Pulmonary Disease	ELX9	I279	Pulmonale Herzkrankheit, nicht näher bezeichnet
Chronic Pulmonary Disease	ELX9	J40	Bronchitis, nicht als akut oder chronisch bezeichnet
Chronic Pulmonary Disease	ELX9	J41	Einfache und schleimig-eitrige chronische Bronchitis
Chronic Pulmonary Disease	ELX9	J42	Nicht näher bezeichnete chronische Bronchitis
Chronic Pulmonary Disease	ELX9	J43	Emphysem
Chronic Pulmonary Disease	ELX9	J44	Sonstige chronische obstruktive Lungenkrankheit
Chronic Pulmonary Disease	ELX9	J45	Asthma bronchiale
Chronic Pulmonary Disease	ELX9	J46	Status asthmaticus
Chronic Pulmonary Disease	ELX9	J47	Bronchiektasen
Chronic Pulmonary Disease	ELX9	J60	Kohlenbergarbeiter-Pneumokoniose
Chronic Pulmonary Disease	ELX9	J61	Pneumokoniose durch Asbest und sonstige anorganische Fasern
Chronic Pulmonary Disease	ELX9	J62	Pneumokoniose durch Quarzstaub
Chronic Pulmonary Disease	ELX9	J63	Pneumokoniose durch sonstige anorganische Stäube
Chronic Pulmonary Disease	ELX9	J64	Nicht näher bezeichnete Pneumokoniose
Chronic Pulmonary Disease	ELX9	J65	Pneumokoniose in Verbindung mit Tuberkulose
Chronic Pulmonary Disease	ELX9	J66	Krankheit der Atemwege durch spezifischen organischen Staub
Chronic Pulmonary Disease	ELX9	J67	Allergische Alveolitis durch organischen Staub
Chronic Pulmonary Disease	ELX9	J684	Chronische Krankheiten der Atmungsorgane durch chemische Substanzen, Gase, Rauch und Dämpfe
Chronic Pulmonary Disease	ELX9	J701	Chronische und sonstige Lungenbeteiligung bei Strahleneinwirkung
Chronic Pulmonary Disease	ELX9	J703	Chronische arzneimittelinduzierte interstitielle Lungenkrankheiten

Supplemental table S3: Parameters of the sensitivity analysis with respect to flail-chest and non-flail injury morphology for the Cox proportional hazard regression of survival to hospital discharge. Covariates were the treatment type (conservative versus surgical stabilisation of rib fractures (SSRF)), sex, age_c in years centred on 67.1 years, Elixhauser score, thorax abbreviated injury scale (AIS) and injury severity score (ISS_c) centred on 16.3 as well as the interactions between SSRF and Age (SSRF:Age_c) and between SSRF and ISS (SSRF:ISS_c). Through centring of age and ISS the hazard ratio for SSRF is interpretable as the hazard ratio for a patient of age 67.1 with ISS 16.3. Given are the hazard ratios (HR), 95% confidence interval of the hazard ratio (95% CI) and p-values. No HR, CI or p-value are reported for thorax AIS in the flail chest cohort, since the presence of a flail chest (S22.5) leads to AIS = 5 in all cases and there was therefore no variability of the covariate within that subgroup.

	Flail chest			Non-flail		
Variable	HR	95% CI	p-value	HR	95% CI	p-value
SSRF	0.137	0.035 – 0.531	**0.004**	0.343	0.089 – 1.312	0.118
Female sex	0.800	0.410 – 1.542	0.498	0.561	0.277 – 1.134	0.109
Age_c	1.052	1.018 – 1.087	**0.002**	1.086	1.039 – 1.134	**< 0.001**
Elixhauser score	1.069	0.969 – 1.179	0.180	1.059	0.934 – 1.202	0.372
Thorax AIS	-	-	-	1.862	0.882 – 3.930	0.103
ISS_c	1.000	0.945 – 1.059	0.989	1.015	0.951 – 1.083	0.654
SSRF:Age_c	1.063	0.986 – 1.146	0.110	1.035	0.949 – 1.129	0.436
SSRF:ISS_c	1.029	0.930 – 1.139	0.579	1.013	0.927 – 1.107	0.775

Supplemental table S4: Parameters of the sensitivity analysis with respect to pulmonary contusion and laceration for the Cox proportional hazard regression of survival to hospital discharge. Covariates were the treatment type (conservative versus surgical stabilisation of rib fractures (SSRF)), sex, age_c in years centred on 67.3 years, Elixhauser score, thorax abbreviated injury scale (AIS) and injury severity score (ISS_c) centred on 16.3 as well as the interactions between SSRF and Age (SSRF:Age_c) and between SSRF and ISS (SSRF:ISS_c). Through centring of age and ISS the hazard ratio for SSRF is interpretable as the hazard ratio for a patient of age 67.1 years with ISS 16.3. Given are the hazard ratios (HR), 95% confidence interval of the hazard ratio (95% CI) and p-values.

	Pulmonary injury present			No pulmonary injury present		
Variable	HR	95% CI	p-value	HR	95% CI	p-value
SSRF	0.293	0.053 – 1.607	0.157	0.193	0.067 – 0.557	**0.002**
Female sex	1.418	0.585 – 3.440	0.440	0.597	0.334 – 1.067	0.082
Age_c	1.079	1.026 – 1.134	**0.003**	1.058	1.024 – 1.092	**< 0.001**
Elixhauser score	1.091	0.938 – 1.268	0.260	1.045	0.955 – 1.144	0.339
Thorax AIS	0.904	0.365 – 2.246	0.828	1.690	0.910 – 3.139	0.096
ISS_c	1.040	0.983 – 1.100	0.175	0.987	0.921 – 1.060	0.735
SSRF:Age_c	1.046	0.943 – 1.161	0.392	1.056	0.991 – 1.126	0.098
SSRF:ISS_c	0.977	0.891 – 1.071	0.618	1.033	0.935 – 1.142	0.519
