# Supplementary material for: The impact of injury severity and age on short-and long-term mortality and hospital length of stay after surgical stabilisation of rib fractures (SSRF): a German population-based propensity-score matched investigation
Source: World J Emerg Surg. 2026 Mar 2;21:21. doi: 10.1186/s13017-026-00682-2 (PMC13059353; doi:10.1186/s13017-026-00682-2)
Supplement: Supplementary file 2 — Supplementary Material 2. [file 13017_2026_682_MOESM2_ESM.pdf]

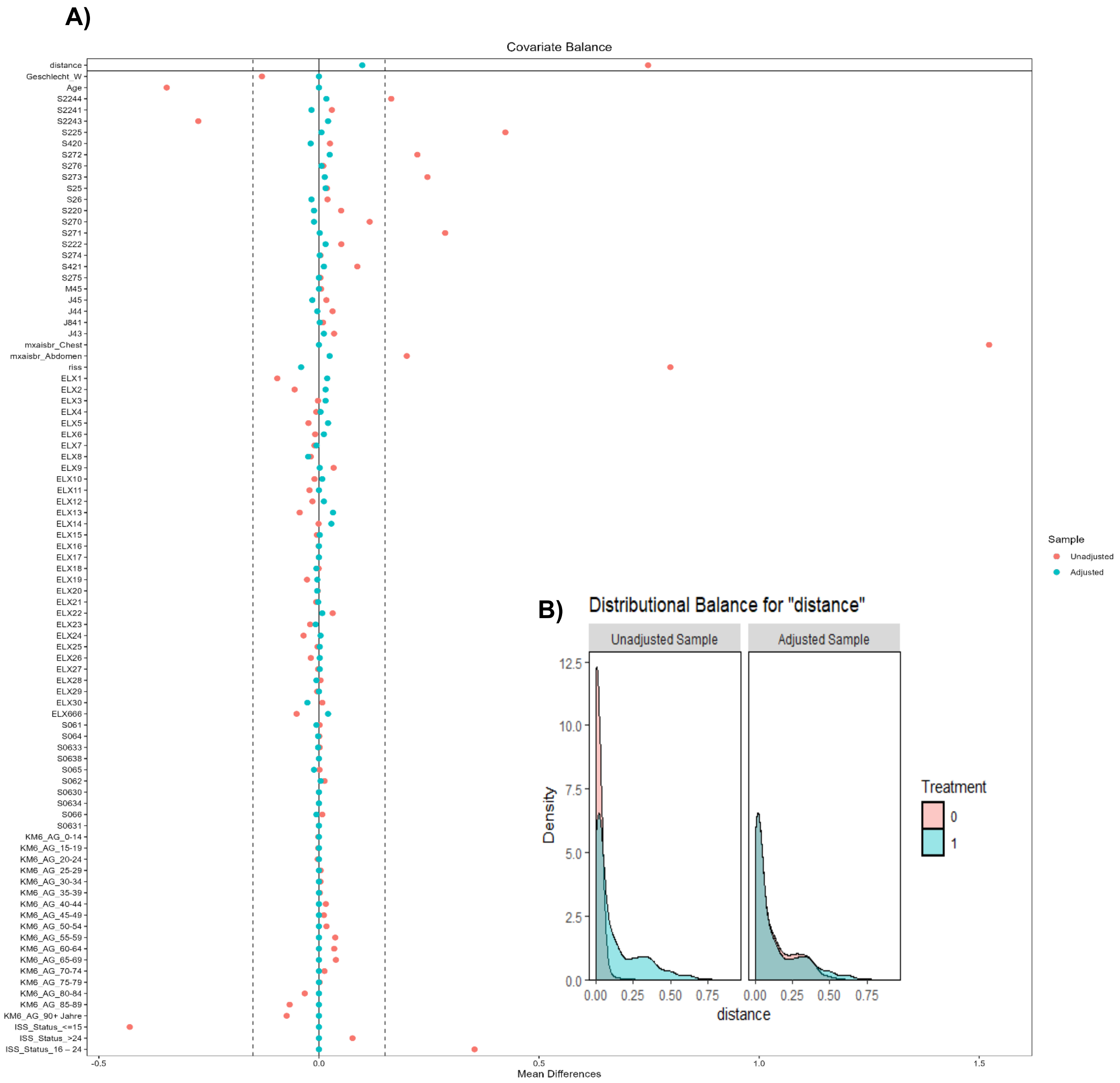

Supplemental figure 1: Shown are plots of Covariate Balance as well as the distribution balance for "distance" in the pre- and post-matching cohorts. A) Covariate Balance plot showing the unadjusted cohort in red and the adjusted cohort in green. B) Plot of distributional balance for "distance" showing the conservative cohort (no SSRF) red and the surgical cohort (SSRF) in green.
